# Synthesis and Biological Activity of Novel *O*-Alkyl Derivatives of Naringenin and Their Oximes

**DOI:** 10.3390/molecules22091485

**Published:** 2017-09-06

**Authors:** Joanna Kozłowska, Bartłomiej Potaniec, Barbara Żarowska, Mirosław Anioł

**Affiliations:** 1 Department of Chemistry, Wrocław University of Environmental and Life Sciences, Norwida 25, 50-375 Wrocław, Poland; b.potaniec@gmail.com (B.P.); miroslaw.aniol@upwr.edu.pl (M.A.); 2 Department of Biotechnology and Food Microbiology, Wrocław University of Environmental and Life Sciences, Chełmońskiego 37/41, 51-630 Wrocław, Poland; barbara.zarowska@upwr.edu.pl

**Keywords:** naringenin, *O*-alkyl derivatives, oximes, antimicrobial activity

## Abstract

*O*-Alkyl derivatives of naringenin (**1a**–**10a**) were prepared from naringenin using the corresponding alkyl iodides and anhydrous potassium carbonate. The resulting products were used to obtain oximes (**1b**–**10b**). All compounds were tested for antimicrobial activity against *Escherichia coli* ATCC10536, *Staphylococcus aureus* DSM799, *Candida albicans* DSM1386, *Alternaria alternata* CBS1526, *Fusarium linii* KB-F1, and *Aspergillus niger* DSM1957. The resulting biological activity was expressed as the increase in optical density (ΔOD). The highest inhibitory effect against *E. coli* ATCC10536 was observed for 7,4′-di-*O*-pentylnaringenin (**8a**), 7-*O*-dodecylnaringenin (**9a**), naringenin oxime (**NG-OX**), 7,4′-di-*O*-pentylnaringenin oxime (**8b**), and 7-*O*-dodecylnaringenin oxime (**9b**) (ΔOD = 0). 7-*O*-dodecylnaringenin oxime (**9b**) also inhibited the growth of *S. aureus* DSM799 (ΔOD = 0.35) and *C. albicans* DSM1386 (ΔOD = 0.22). The growth of *A. alternata* CBS1526 was inhibited as a result of the action of 7,4′-di-*O*-methylnaringenin (**2a**), 7-*O*-ethylnaringenin (**4a**), 7,4′-di-*O*-ethylnaringenin (**5a**), 5,7,4′-tri-*O*-ethylnaringenin (**6a**), 7,4′-di-*O*-pentylnaringenin (**8a**), and 7-*O*-dodecylnaringenin (**9a**) (ΔOD in the range of 0.49–0.42) in comparison to that of the control culture (ΔOD = 1.87). In the case of *F. linii* KB-F1, naringenin (**NG**), 7,4′-di-*O*-dodecylnaringenin (**10a**), 7-*O*-dodecylnaringenin oxime (**9b**), and 7,4′-di-*O*-dodecylnaringenin oxime (**10b**) showed the strongest effect (ΔOD = 0). 7,4′-Di-*O*-pentylnaringenin (**8a**) and naringenin oxime (**NG-OX**) hindered the growth of *A. niger* DSM1957 (ΔOD = 0).

## 1. Introduction

Flavonoids are polyphenolic compounds, which are widespread in plants and food. This group comprises flavones, flavanones, flavonols, isoflavones, anthocyanidins and flavanols [1]. In plants, flavonoids usually occur in glycoside form [2,3]. Naringin is the 7-rhamno-glucoside of naringenin, which is one of the most popular flavonoids present in citrus fruits. The presence of such glycoside derivatives of flavonoids is responsible for the bitter taste of grapefruit juice [4].

**Figure 1:**
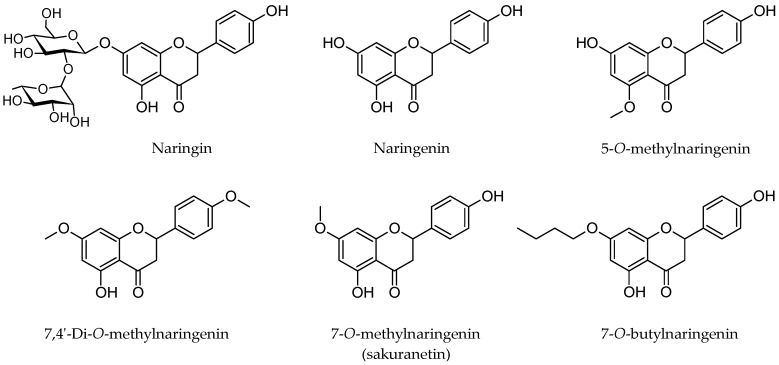
Structures of naringin, naringenin and naringenin derivatives.

In the present paper, the most interesting substrate was naringenin (4′,5,7-trihydroxyflavanone), which possesses a wide spectrum of biological activities including antibacterial, antifungal, antioxidant, and anticancer activities [5,6]. Currently, there are known *O*-alkyl derivatives of naringenin containing methyl and ethyl groups attached to ring A and B. Naringenin and ether derivatives of naringenin were observed in plant extracts of the Boraginaceae family. In particular, 5-*O*-methylnaringenin, 7,4′-di-*O*-methylnaringenin and sakuranetin (7-*O*-methylnaringenin) (Figure 1) were isolated from *Cordia globosa*, *Echiochilon fruticosum*, *Heliotropium indicum*, *Heliotropium stenophyllum* and *Corymbia torelliana* [7,8]. Sakuranetin, which is present in rice plants, is a natural phytoalexin and provides effective protection against damage caused by microorganisms [9]. Moreover, it possesses anti-inflammatory activity and prevents vascular and parenchymal changes [10]. Derivatives with a benzyl group at the C-7 position regulate apoptosis in human colorectal carcinoma (RKO) cells as a result of the intracellular production of reactive oxygen species (ROS) [11]. Furthermore, 7-*O*-butylnaringenin exhibits antibacterial activity against methicillin-resistant *Staphylococcus aureus* (MRSA), which is one of the most important pathogens in hospitals [12].

Oxime derivatives possess very promising biological properties, e.g., antifungal [13,14], antioxidant [15,16,17], anticancer [18,19,20] and antiplatelet activities [21]. Modified flavonoids from *Kaempferia parviflora* exhibit antiproliferative activity toward epidermoid carcinoma of oral cavity (KB) and human small cell lung cancer (NCI-H187) cell lines about seven times higher than the analogue without the =NOH group [22]. Moreover, cytotoxicity assays performed on rat pheochromocytoma cell lines (PC-12), and additionally on human colon (HT-29) and breast (MCF-7) cancer cell lines, show that the oxime group increases the inhibitory effect for proliferation [18].

Current knowledge of *O*-alkyl derivatives reveals the diversity of their biological activities [7,8,9,10,11,12,23]. Our study was focused on the efficient synthesis of novel *O*-alkyl derivatives of naringenin and their oximes, which have not been mentioned in the literature. In the presented study, the focus is on their antimicrobial activity against different strains of bacteria and fungi. Our research proved that elongation of the *O*-alkyl chain at the C-7 and C-4′ positions in naringenin leads to a significant increase in the biological activity of the obtained compounds. In addition, our studies allow the determination of the influence of introduction of the oxime group on the growth of some pathogenic strains of bacteria and fungi and compared it with the results for *O*-alkyl derivatives.

## 2. Results and Discussion

**Scheme 1:**
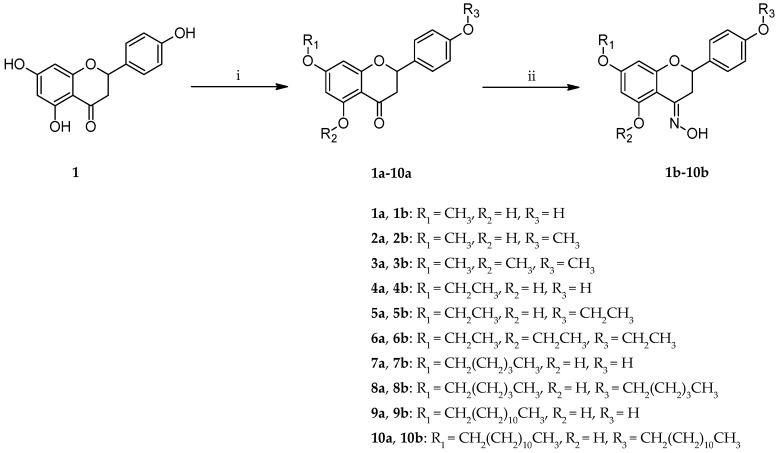
Synthesis of *O*-alkyl derivatives **1a**–**10a** and their oximes **1b**–**10b**; Reaction conditions: (**i**) alkyl iodide, (CH_3_)_2_CO or DMF, K_2_CO_3_, r.t., 24–96 h; (**ii**) NH_2_OH·HCl, CH_3_COONa, EtOH, 45 °C, 24–96 h.

*O*-Alkyl derivatives were obtained by a one-step synthesis from naringenin using the appropriate alkyl iodide in the presence of potassium carbonate (**1a**–**10a**). First, reactions were performed in anhydrous acetone at room temperature for 24–96 h, which afforded a mixture of 7-*O*-alkyl- (**1a**, **4a**, **7a**, **9a**) and 7,4′-di-*O*-alkylnaringenin (**2a**, **5a**, **8a**, **10a**). When using *N*,*N*-dimethylformamide (DMF) as the solvent, and after 7 h of reaction, 5,7,4′-tri-*O*-alkylnaringenin (**3a**, **6a**) was obtained. In the second step, which involved the reaction with hydrochloride hydroxylamine and anhydrous sodium acetate in ethanol, naringenin analogues were transformed into oximes (**1b**–**10b**) (Scheme 1). All crude products were purified by column chromatography, and their purity was analysed by high-performance liquid chromatography (HPLC).

The structures of these compounds were confirmed by ^1^H- and ^13^C-NMR. Analysis of signals in ^1^H-NMR spectrum of *O*-alkyl derivatives allowed to identify the methyl, ethyl, pentyl, and dodecyl groups attached to the 5, 7 and 4′ positions in naringenin (**1a**–**10a**). In the case of sakuranetin (7-*O*-methylnaringenin, **1a**), signals at 12.02 ppm attributed to the hydroxyl moiety attached to the C-5 position, and at 5.21 ppm due to the substituent at C-4′ were observed. In the case of 7,4′-di-*O*-methylnaringenin (**2a**), only one singlet at 12.03 ppm was observed, which confirmed the substitution of methyl groups at the C-4′ and C-7 positions. The shift of the signal due to the hydroxyl moiety at C-5 to the 12.03 ppm is caused by the formation of intramolecular hydrogen bonding with the carbonyl group. Moreover, this bonding has an effect on the low reactivity of this group with alkyl iodide [24]. In view of the thermodynamic equilibrium between flavanones and chalcones, signals from H-2 at 5.36 ppm (dd, *J* = 13.2, 3.0 Hz), H-3_a_ at 3.09 ppm (dd, *J* = 17.2, 13.2 Hz) and H-3_b_ at 2.79 ppm (dd, *J* = 17.2, 3.0 Hz) confirmed that the obtained derivatives had a flavanone skeleton. Furthermore, the signal at 196.23 ppm in the ^13^C-NMR spectrum provides information about the presence of a carbonyl group in each product. In the case of oxime derivatives (**1b**–**10b**), a peak from the =NOH group at 11.03–10.89 ppm was observed. Besides, the downshift from 196.21 ppm to 154.85 ppm in the ^13^C-NMR spectra indicated the replacement of the carbonyl group with the oxime moiety.

In our study, the biological properties of the obtained derivatives were verified. These studies were performed to describe the inhibitory effect of the *O*-alkyl derivatives (**1a**–**10a**) (Table 1) and their oximes (**1b**–**10b**) (Table 2) on two strains of bacteria and four strains of fungi.

**Figure 2:**
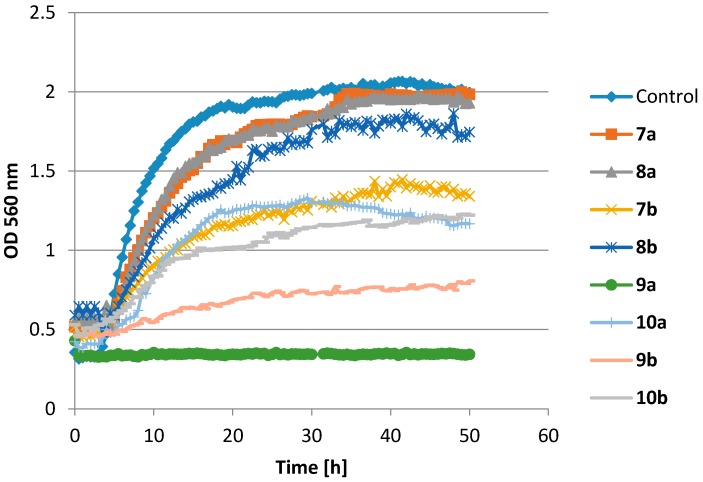
The effect of action of *O*-alkyl derivatives of naringenin (**7a**–**10a**) and their oximes (**7b**–**10b**) on the growth of *S. aureus* DSM799.

Susceptibility to the tested compounds was an individual feature of each strain. In the case of 7,4′-di-*O*-pentylnaringenin (**8a**) complete growth inhibition of *E. coli* ATCC10536 and *A. niger* DSM1957 was observed. Furthermore, compound **9a** totally inhibited the growth of *E. coli* ATCC10536. In comparison to naringenin, **10a** showed a 6 times stronger restriction of *E. coli* ATCC10536 growth and considerable reduction of the adaptive phase from 15 to 3 h. Moreover, this compound showed full growth inhibition of *F. linii* KB-F1. Lee et al. reported that 7-*O*-butylnaringenin displayed antimicrobial activity against methicillin-resistant *S. aureus* (MRSA). The action of this derivative was compared with that of naturally occurring flavonoids—quercetin and naringenin—and has been described as Minimal Inhibitory Concentration (MIC) [12]. In our research, **9a**, which has a dodecyl group attached to the C-7 position in naringenin, exhibited the best inhibitory effect against *S. aureus* DSM799 among all the tested *O*-alkyl derivatives (Figure 2). The results of our studies suggest that elongation of the alkyl chain improves the inhibitory effect on *S. aureus* strain. The only compound that limited the differentiation of *C. albicans* DSM1386 was **6a**. Satisfying results were observed for *A. alternata* CBS1526 in the presence of compounds **2a**, **4a**–**6a**, **8a** and **9a**.

Biological assays performed on oxime derivatives allowed to evaluate the effect of introduction of the =NOH group on antimicrobial activity.

**Figure 3:**
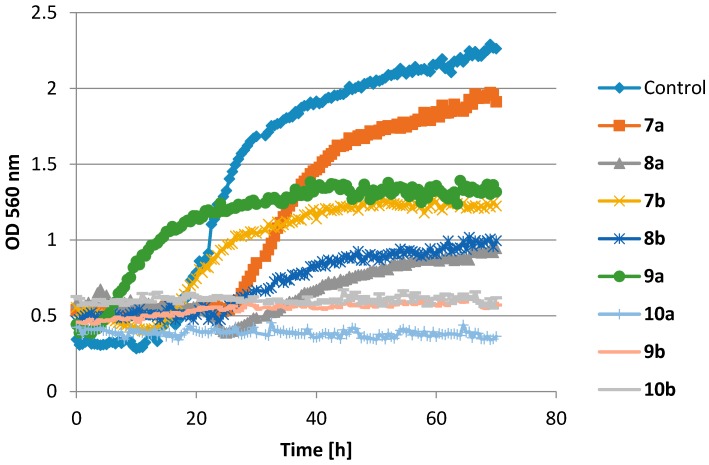
The effect of action of *O*-alkyl derivatives of naringenin (**7a**–**10a**) and their oximes (**7b**–**10b**) on the growth of *F. linii* KB-F1.

The strongest inhibitory effect was observed for compounds **8b** and **9b** in *E. coli* ATCC10536 culture. Moreover, naringenin oxime completely prevented the growth of this strain of bacteria and *A. niger* DSM1957. Compounds **2b** and **5b** also showed a strong inhibitory effect against this filamentous fungus. Derivative **9b** also hindered *A. niger* DSM1957 growth and, additionally, prolonged the adaptive phase. Total inhibition of *F. linii* KB-F1 growth was achieved by the action of oximes **9b** and **10b** (Figure 3).

In the case of *S. aureus* DSM799, 7-*O*-dodecylnaringenin oxime (**9b**) had an inhibitory effect about 3 times stronger than 7-*O*-dodecylnaringenin (**9a**). Interestingly, the addition of **9b** to the *S. aureus* DSM799 culture resulted in a shorter adaptive phase than that with **9a** (Figure 2). Feng et al. described that oxime derivatives with long alkyl chains attached to 5,7-dihydroxy-4-chromanone show strong antimicrobial activity against *S. aureus* (MRSA). It is worth mentioning that the substitution of the =NOH group with a methyl or benzyl group is not favoured [25].

Yenjai et al. reported that extracts from *Kaempferia parviflora* contain various *O*-methyl derivatives of flavone. Furthermore, an investigation performed by a scientific group in Thailand showed that the introduction of the =NOH group instead of carbonyl enhances the biological properties of the modified compounds. The oxime derivative with two hydroxyl moieties at the C-5 and C-7 positions exhibited antifungal activity against *C. albicans* with an IC_50_ value of 48.98 µg/mL [22,26]. A similar trend was observed in our study. In the case of *C. albicans* DSM1386, a stronger inhibitory effect of **9b** than that of **9a** was noticed. This proves that the introduction of the =NOH group significantly enhances the antimicrobial properties of this derivative.

Isosakuranetin (4′-*O*-methylnaringenin), obtained from flowers of *Chromolaena odorata*, exhibited moderate activity against *Mycobacterium tuberculosis* with the MIC value of 174.8 µM [27]. Moreover, it decreased growth of *Helicobacter pylori* but hardly inhibited the urease activity of this strain of bacteria [28]. In our investigation, the isomer of isosakuranetin—sakuranetin (**1a**) exhibited a satisfactory inhibitory effect. This effect was about 2 times stronger than that of naringenin for *E. coli* ATCC10536 (∆OD = 0.75) and *A. alternata* CBS1526 (∆OD = 0.72) culture, but was not as strong as that of compounds **8a** and **9a** (∆OD = 0). Our studies confirmed that elongation of the hydrophobic chain increased the antimicrobial activities.

The therapeutic potential of oxime derivatives of flavonoids has not been well studied. Ilboudo et al. reported that the oxime obtained by chemical modification of the butanolic fraction from *Mentha piperita* exhibited stronger antifungal activity against *Phoma sorghina* and *Fusarium moniliforme* [29]. In our investigation, the replacement of carbonyl with the oxime group had a significant influence on not only *F. linii* KB-F1 (**9a**—∆OD = 0.98, **9b**—∆OD = 0), but also *C. albicans* DSM1386 (**9a**—∆OD = 0.97, **9b**—∆OD = 0.22) and *A. niger* DSM1957 growth (**9a**—∆OD = 0.96, **9b**—∆OD = 0.58).

## 3. Materials and Methods

### 3.1. Chemicals

Naringenin, iodomethane, iodoethane, 1-iodopentane and 1-iodododecane were purchased from Sigma-Aldrich Chemical Co. (Steinheim, Germany), hydroxylamine hydrochloride from LOBA Feinchemie GmbH (Fischamed, Austria), anhydrous sodium acetate and potassium carbonate from Chempur (Piekary Śląskie, Poland). Anhydrous solvents were prepared according to standard procedures. All organic solvents were of analytical grade.

### 3.2. Analysis

The reaction progress was analysed by thin layer chromatography (TLC) on silica gel-coated aluminium plates with fluorescent indicator (DC-Alufolien, Kieselgel 60 F_254_; Merck, Darmstadt, Germany). Products were detected by spraying the plates with a solution of 1% Ce(SO_4_)_2_ and 2% H_3_[P(Mo_3_O_10_)_4_] in 5% H_2_SO_4_ and subsequently visualised by heating. Crude products were purified by liquid column chromatography using silica gel (Kieselgel 60, 230–400 mesh, Merck). The purity of the products was analysed by HPLC on a Waters 2690 (Milford, MA, USA) with Photodiode Array Detector Waters 996. The HPLC apparatus was equipped with a reverse-phase C-18 column (Phenomenex, Torrance, CA, United States, Kinetex 5u XB-C18 100A, 250 mm × 4.6 mm), which was thermostated at 28 °C, and analysed samples were kept at 12 °C. The mobile phase consisted of two eluents: A—1% HCOOH in MeCN and B—1% HCOOH in H_2_O. Elution gradient was started from 55% of eluent A to 45% of eluent B over 21 min. A flow rate of 1.5 mL/min was used. The samples were dissolved in methanol.

Nuclear magnetic resonance (NMR) analysis was performed to elucidate the structure of the received compounds. ^1^H-NMR and ^13^C-NMR spectra were recorded on a Bruker Avance^TM^600 MHz spectrometer (Bruker, Billerica, MA, USA) with acetone-d6, chloroform-d, and dimethyl sulfoxide-d6 as solvents (Supplementary Materials, Figures S1–S40).

Positive-ion HR ESI-MS spectra were measured on a Bruker ESI-Q-TOF Maxis Impact Mass Spectrometer (Bruker, Billerica, MA, USA). The direct infusion of ESI-MS parameters: the mass spectrometer was operated in positive ion mode with the potential between the spray needle and the orifice 3.5 kV, nebulizer pressure of 0.4 bar, and a drying gas flow rate of 3.0 L/min at 200 °C. The sample flow rate was 3.0 µL/min. Ionization mass spectra were collected at the ranges *m*/*z* 50–1250.

UV spectra were recorded in methanol on a Cintra 303 spectrophotometer (GBC, Braeside, Australia). Melting points (uncorrected) were determined on a Boetius apparatus (Jena, Germany).

### 3.3. Synthesis of O-Alkyl Derivatives of Naringenin

#### 3.3.1. Synthesis of Mono- (**1a**, **4a**, **7a**, **9a**) and Di-*O*-alkyl Derivatives of Naringenin (**2a**, **5a**, **8a**, **10a**)

Anhydrous potassium carbonate (11.02 mmol) and the relevant alkyl iodide (36.73 mmol) were added to naringenin (7.35 mmol) dissolved in anhydrous acetone (20 mL). Reactions were performed for 24–96 h at room temperature. Then, the organic solvent was evaporated, and the resultant reaction mixture was treated with a saturated solution of sodium chloride (40 mL) and extracted with diethyl ether (3 × 50 mL). The organic solvent was dried over sodium sulphate and concentrated on a vacuum evaporator. The crude products were separated by column chromatography.

#### 3.3.2. Synthesis of Tri-*O*-alkyl Derivatives of Naringenin (**3a**, **6a**)

Anhydrous potassium carbonate (22.04 mmol) and the appropriate alkyl iodide (22.04 mmol) were added to naringenin (3.67 mmol) dissolved in DMF (10 mL). Reactions were performed for 7–24 h at room temperature. Then, 1 M HCl was added dropwise until pH 7 was reached. The resultant mixture was extracted with methylene chloride (3 × 50 mL). The organic solvent was dried over sodium sulphate and concentrated on a vacuum evaporator. The crude products were isolated by column chromatography.

*7-O-Methylnaringenin* (**1a**), Yield 39.6% (2.08 g), yellow powder, m.p. 144 °C, lit. 143–144 °C [30]. ^1^H-NMR (600 MHz, CDCl_3_) δ (ppm): 12.02 (s, 1H, OH-5), 7.36–7.30 (m, 2H, AA′BB′, H-2′, H-6′), 6.91–6.86 (m, 2H, AA′BB′, H-3′, H-5′), 6.07 (d, *J* = 2.2 Hz, 1H, H-6), 6.04 (d, *J* = 2.2 Hz, 1H, H-8), 5.36 (dd, *J* = 13.2, 3.0 Hz, 1H, H-2), 5.21 (s, 1H, OH-4′), 3.81 (s, 3H, -CH_3_), 3.09 (dd, *J* = 17.2, 13.2 Hz, 1H, H-3_a_), 2.79 (dd, *J* = 17.2, 3.0 Hz, 1H, H-3_b_). ^13^C-NMR (150 MHz, CDCl_3_) δ (ppm): 196.21 (C=O), 168.15, 164.25, 163.02, 156.28, 130.67, 128.11, 115.82, 103.27, 95.26, 94.41, 79.11, 55.85, 43.34. HR ESI-MS *m*/*z* calculated for C_16_H_14_O_5_ [M + H]^+^ 287.0914, found [M + H]^+^ 287.0917, lit. HR ESI-MS *m*/*z* calculated for C_16_H_13_O_5_ [M − H]^−^ 285.0763, found [M − H]^−^ 285.0771 [8].

*7,4*′*-Di-O-methylnaringenin* (**2a**), Yield 42.3% (2.33 g), pale yellow powder, m.p. 112–115 °C, lit. 114–115 °C [31]. ^1^H-NMR (600 MHz, CDCl_3_) δ (ppm): 12.03 (s, 1H, OH-5), 7.41–7.35 (m, 2H, AA′BB′, H-2′,H-6′), 6.98–6.91 (m, 2H, AA′BB′, H-3′, H-5′), 6.07 (d, *J* = 2.3 Hz, 1H, H-6), 6.04 (d, *J* = 2.3 Hz, 1H, H-8), 5.36 (dd, *J* = 13.1, 3.0 Hz, 1H, H-2), 3.83 (s, 3H, -CH_3_), 3.80 (s, 3H, -CH_3_), 3.10 (dd, *J* = 17.2, 13.1 Hz, 1H, H-3_a_), 2.79 (dd, *J* = 17.2, 3.0 Hz, 1H, H-3_b_). ^13^C-NMR (150 MHz, CDCl_3_) δ (ppm): 196.16 (C=O), 168.09, 164.26, 163.03, 160.18, 130.51, 127.87, 114.36, 103.27, 95.22, 94.36, 79.15, 55.82, 55.51, 43.34. HR ESI-MS *m*/*z* calculated for C_17_H_16_O_5_ [M + H]^+^ 301.1071, found [M + H]^+^ 301.1086, lit. HR ESI-MS *m*/*z* calculated for C_17_H_16_O_5_Na^+^ [M + Na]^+^ 323.0890, found [M + Na]^+^ 323.0873 [31].

*5,7,4*′*-Tri-O-methylnaringenin* (**3a**), Yield 72.3% (0.840 g), white powder, m.p. 126–129 °C, lit. 120–122 °C [22]. ^1^H-NMR (600 MHz, CDCl_3_) δ (ppm): 7.41–7.35 (m, 2H, AA′BB′, H-2′, H-6′), 6.97–6.91 (m, 2H, AA′BB′, H-3′ H-5′), 6.14 (d, *J* = 2.3 Hz, 1H, H-6), 6.09 (d, *J* = 2.3 Hz, 1H, H-8), 5.35 (dd, *J* = 13.2, 2.9 Hz, 1H, H-2), 3.89 (s, 3H, -CH_3_), 3.83 (s, 3H, -CH_3_), 3.81 (s, 3H, -CH_3_), 3.03 (dd, *J* = 16.5, 2.9 Hz, 1H, H-3_a_), 2.76 (dd, *J* = 16.5, 2.9 Hz, 1H, H-3_b_). ^13^C-NMR (150 MHz, CDCl_3_) δ (ppm): 189.59 (C=O), 166.06, 165.20, 162.40, 160.03, 130.92, 127.84, 114.27, 106.12, 93.66, 93.25, 79.12, 56.30, 55.72, 55.49, 45.54. HR ESI-MS *m*/*z* calculated for C_18_H_18_O_5_ [M + H]^+^ 315.1227, found [M + H]^+^ 315.1229, lit. HR ESI-MS *m*/*z* calculated for C_18_H_18_O_5_Na^+^ [M + Na]^+^ 337.1052, found [M + Na]^+^ 337.1052 [22].

*7-O-Ethylnaringenin* (**4a**), Yield 67.8% (1.50 g), yellow powder, m.p. 132–134 °C, lit. 130–131 °C [32]. ^1^H-NMR (600 MHz, CDCl_3_) δ (ppm): 12.01 (s, 1H, OH-5), 7.32 (d, *J* = 8.1 Hz, 2H, H-2′, H-6′), 6.88 (d, *J* = 8.1 Hz, 2H, H-3′, H-5′), 6.06 (d, *J* = 2.3 Hz, 1H, H-6), 6.03 (d, *J* = 2.3 Hz, 1H, H-8), 5.58 (s, 1H, OH-4′), 5.34 (dd, *J* = 12.9, 3.0 Hz, 1H, H-2), 4.03 (q, *J* = 7.0 Hz, 2H, -CH_2_-), 3.09 (dd, *J* = 17.2, 12.9 Hz, 1H, H-3_a_), 2.78 (dd, *J* = 17.2, 3.0 Hz, 1H, H-3_b_), 1.40 (t, *J* = 7.0 Hz, 3H, -CH_3_). ^13^C-NMR (150 MHz, CDCl_3_) δ (ppm): 196.17 (C=O), 167.61, 164.21, 163.03, 156.26, 130.72, 128.11, 115.81, 103.17, 95.69, 94.76, 79.06, 64.26, 43.31, 14.67. HR ESI-MS *m*/*z* calculated for C_17_H_16_O_5_ [M + H]^+^ 301.1071, found [M + H]^+^ 301.1081.

*7,4*′*-Di-O-ethylnaringenin* (**5a**), Yield 23.5% (0.566 g), white powder, m.p. 97–102 °C, lit. 97–98 °C [32]. ^1^H-NMR (600 MHz, CDCl_3_) δ (ppm): 12.02 (s, 1H, OH-5), 7.39–7.33 (m, 2H, AA′BB′, H-2′, H-6′), 6.97–6.91 (m, 2H, AA′BB′, H-3′, H-5′), 6.05 (d, *J* = 2.3 Hz, 1H, H-6), 6.02 (d, *J* = 2.3 Hz, 1H, H-8), 5.35 (dd, *J* = 13.0, 3.0 Hz, 1H, H-2), 4.06 (q, *J* = 7.0 Hz, 2H, -CH_2_-), 4.03 (q, *J* = 7.0 Hz, 2H, -CH_2_-), 3.09 (dd, *J* = 17.1, 13.0 Hz, 1H, H-3_a_), 2.78 (dd, *J* = 17.1, 3.0 Hz, 1H, H-3_b_), 1.43 (t, *J* = 7.0 Hz, 3H, -CH_3_), 1.40 (t, *J* = 7.0 Hz, 3H, -CH_3_). ^13^C-NMR (150 MHz, CDCl_3_) δ [ppm]: 196.12 (C=O), 167.51, 164.23, 163.04, 159.55, 130.38, 127.85, 114.88, 103.18, 95.63, 94.69, 79.15, 64.21, 63.71, 43.34, 14.93, 14.67. HR ESI-MS *m*/*z* calculated for C_19_H_20_O_5_ [M + H]^+^ 329.1386, found [M + H]^+^ 329.1400.

*5,7,4*′*-Tri-O-ethylnaringenin* (**6a**), Yield 58.9% (0.385 g), white powder, m.p. 117–120 °C. ^1^H-NMR (600 MHz, CDCl_3_) δ (ppm): 7.39–7.34 (m, 2H, AA′BB′, H-2′, H-6′), 6.95–6.89 (m, 2H, AA′BB′, H-3′, H-5′), 6.10 (d, *J* = 2.2 Hz, 1H, H-6), 6.06 (d, *J* = 2.2 Hz, 1H, H-8), 5.33 (dd, *J* = 13.4, 2.9 Hz, 1H, H-2), 4.11–4.07 (m, 2H, -CH_2_-), 4.07–4.00 (m, 4H, 2x-CH_2_-), 3.02 (dd, *J* = 16.5, 13.4 Hz, 1H, H-3a), 2.74 (dd, *J* = 16.5, 2.9 Hz, 1H, H-3b), 1.51 (t, *J* = 7.0 Hz, 3H, -CH_3_), 1.42 (t, *J* = 7.0 Hz, 3H, -CH_3_) 1.41 (t, *J* = 7.0 Hz, 3H, -CH_3_). ^13^C-NMR (150 MHz, CDCl_3_) δ (ppm): 189.48 (C=O), 165.33, 165.14, 161.72, 159.38, 130.86, 127.82, 114.80, 106.15, 94.38, 93.93, 79.13, 64.74, 64.01, 63.68, 45.66, 14.93, 14.72, 14.69. HR ESI-MS *m*/*z* calculated for C_21_H_24_O_5_ [M + H]^+^ 357.1697, found [M + H]^+^ 357.1699.

*7-O-Pentylnaringenin* (**7a**), Yield 72.3% (1.81 g), yellow powder, m.p. 110–113 °C. ^1^H-NMR (600 MHz, CDCl_3_) δ (ppm): 12.01 (s, 1H, OH-5), 7.35–7.31 (m, 2H, AA′BB′, H-2′, H-6′), 6.91–6.85 (m, 2H, AA′BB′, H-3′, H-5′), 6.09 (d, *J* = 2.4 Hz, 1H, H-6), 6.07 (d, *J* = 2.4 Hz, 1H, H-8), 5.35 (dd, *J* = 13.0, 3.0 Hz, 1H, H-2), 5.19 (s, 1H, OH-4′), 3.96 (t, *J* = 6.6 Hz, 2H, -CH_2_-), 3.08 (dd, *J* = 17.1, 13.0 Hz, 1H, H-3_a_), 2.78 (dd, *J* = 17.1, 3.0 Hz, 1H, H-3_b_), 1.77 (p, *J* = 6.9 Hz, 2H, -CH_2_-), 1.44–1.33 (m, 4H, 2x-CH_2_-), 0.92 (t, *J* = 7.1 Hz, 3H, -CH_3_). ^13^C-NMR (150 MHz, CDCl_3_) δ (ppm): 196.12 (C=O), 167.81, 164.21, 163.01, 156.24, 130.77, 128.10, 115.81, 103.14, 95.72, 94.76, 79.05, 68.72, 43.33, 28.73, 28.17, 22.50, 14.12. HR ESI-MS *m*/*z* calculated for C_20_H_22_O_5_ [M + H]^+^ 343.1540, found [M + H]^+^ 343.1547.

*7,4*′*-Di-O-pentylnaringenin* (**8a**), Yield 30.8% (0.932 g), pale yellow powder, m.p. 67–70 °C. ^1^H-NMR (600 MHz, CDCl_3_) δ (ppm): 12.02 (s, 1H, OH-5), 7.38–7.33 (m, 2H, AA′BB′, H-2′, H-6′), 6.96–6.92 (m, 2H, AA′BB′, H-3′, H-5′), 6.05 (d, *J* = 2.2 Hz, 1H, H-6), 6.02 (d, *J* = 2.2 Hz, 1H, H-8), 5.35 (dd, *J* = 13.0, 3.0 Hz, 1H, H-2), 3.97 (t, *J* = 6.6 Hz, 2H, -CH_2_-), 3.95 (t, *J* = 6.6 Hz, 2H, -CH_2_-), 3.09 (dd, *J* = 17.2, 13.0 Hz, 1H, H-3_a_), 2.77 (dd, *J* = 17.2, 3.0 Hz, 1H, H-3_b_), 1.84–1.73 (m, 4H, 2x-CH_2_-), 1.49–1.32 (m, 8H, 4x-CH_2_-), 0.94 (t, *J* = 7.4 Hz, 3H, -CH_3_), 0.92 (t, *J* = 7.4 Hz, 3H, -CH_3_). ^13^C-NMR (150 MHz, CDCl_3_) δ (ppm): 196.10 (C=O), 167.72, 164.22, 163.05, 159.75, 130.33, 127.82, 114.89, 103.15, 95.66, 94.70, 79.14, 68.68, 68.26, 43.34, 29.05, 28.73, 28.32, 28.17, 22.59, 22.51, 14.16, 14.12. HR ESI-MS *m*/*z* calculated for C_25_H_32_O_5_ [M + H]^+^ 413.2323, found [M + H]^+^ 413.2345.

*7-O-Dodecylnaringenin* (**9a**), Yield 70.2% (1.14 g), pale yellow powder, m.p. 101–105 °C. ^1^H-NMR (600 MHz, CDCl_3_) δ (ppm): 12.01 (s, 1H, OH-5), 7.36–7.31 (m, 2H, AA′BB′, H-2′, H-6′), 6.91–6.85 (m, 2H, AA′BB′, H-3′, H-5′), 6.05 (d, *J* = 2.2 Hz, 1H, H-6), 6.03 (d, *J* = 2.2 Hz, 1H, H-8), 5.35 (dd, *J* = 13.0, 3.0 Hz, 1H, H-2), 5.00 (s, 1H, OH-4′), 3.95 (t, *J* = 6.6 Hz, 2H, -CH_2_-), 3.08 (dd, *J* = 17.1, 13.0 Hz, 1H, H-3_a_), 2.78 (dd, *J* = 17.1, 3.0 Hz, 1H, H-3_b_), 1.76 (p, *J* = 6.6 Hz, 2H, -CH_2_-), 1.44–1.38 (m, 2H, -CH_2_-), 1.34–1.20 (m, 16H, 8x-CH_2_-), 0.88 (t, *J* = 7.0 Hz, 3H, -CH_3_). ^13^C-NMR (150 MHz, CDCl_3_) δ (ppm): 196.04 (C=O), 167.77, 164.22, 162.98, 156.20, 130.83, 128.10, 115.80, 103.14, 95.71, 94.74, 79.05, 68.73, 43.37, 32.06, 29.79, 29.77, 29.72, 29.67, 29.49, 29.43, 29.03, 26.03, 22.84, 14.28. HR ESI-MS *m*/*z* calculated for C_27_H_36_O_5_ [M + H]^+^ 441.2636, found [M + H]^+^ 441.2654.

*7,4*′*-Di-O-dodecylnaringenin* (**10a**), Yield 20.5% (0.457 g), white powder, m.p. 60–62 °C. ^1^H-NMR (600 MHz, CDCl_3_) δ (ppm): 12.02 (s, 1H, OH-5), 7.38–7.34 (m, 2H, AA′BB′, H-2′, H-6′), 6.96–6.91 (m, 2H, AA′BB′, H-3′, H-5′), 6.05 (d, *J* = 2.2 Hz, 1H, H-6), 6.02 (d, *J* = 2.2 Hz, 1H, H-8), 5.35 (dd, *J* = 13.0, 3.0 Hz, 1H, H-2), 3.97 (t, *J* = 6.6 Hz, 2H, -CH_2_-), 3.95 (t, *J* = 6.6 Hz, 2H, -CH_2_-), 3.09 (dd, *J* = 17.2, 13.0 Hz, 1H, H-3_a_), 2.78 (dd, *J* = 17.2, 3.0 Hz, 1H, H-3_b_), 1.83–1.71 (m, 4H, 2x-CH_2_-), 1.48–1.38 (m, 4H, 2x-CH_2_-), 1.34–1.21 (m, 32H, 16x-CH_2_-), 0.88 (t, *J* = 7.0 Hz, 3H, -CH_3_), 0.88 (t, *J* = 7.0 Hz, 3H, -CH_3_). ^13^C-NMR (150 MHz, CDCl_3_) δ (ppm): 196.12 (C=O), 167.73, 164.22, 163.04, 159.76, 130.32, 127.83, 114.90, 103.15, 95.66, 94.71, 79.16, 68.71, 68.28, 43.35, 32.07, 29.81, 29.79, 29.77, 29.75, 29.73, 29.72, 29.68, 29.54, 29.50, 29.43, 29.36, 29.04, 26.18, 26.03, 22.84, 14.28. HR ESI-MS *m*/*z* calculated for C_39_H_60_O_5_ [M + H]^+^ 609.4514, found [M + H]^+^ 609.4499.

### 3.4. Synthesis of Oximes *(**1b**–**10b**)*

Hydroxylamine hydrochloride (1.60 mmol) and anhydrous sodium acetate (1.60 mmol) were added to the *O*-alkyl derivative of naringenin (1.06 mmol) (**1a**–**10a**) dissolved in anhydrous ethanol (10 mL). Reaction was performed on magnetic stirrer at 40–50 °C. Then, the mixture was poured into ice water and the precipitated crystals were collected. The crude products were purified by column chromatography. In some cases, washing with cold water was sufficient to obtain the desired product with satisfactory purity.

*7-O-Methylnaringenin oxime* (**1b**), Yield 99.4% (0.525 g), white powder, m.p. 195–200 °C. ^1^H-NMR (600 MHz, acetone-*d*_6_) δ (ppm): 11.03 (s, 1H, NOH), 10.40 (s, 1H, OH-5), 8.48 (s, 1H, OH-4′), 7.42–7.34 (m, 2H, AA′BB′, H-2′, H-6′), 6.94–6.86 (m, 2H, AA′BB′, H-3′, H-5′), 6.05 (d, *J* = 2.5 Hz, 1H, H-6), 6.04 (d, *J* = 2.5 Hz, 1H, H-8), 5.08 (dd, *J* = 12.0, 3.1 Hz, 1H, H-2), 3.76 (s, 3H, -CH_3_), 3.46 (dd, *J* = 17.1, 3.1 Hz, 1H, H-3_a_), 2.79 (dd, *J* = 17.1, 12.0 Hz, 1H, H-3_b_). ^13^C-NMR (150 MHz, acetone-*d*_6_) δ (ppm): 163.47, 160.65, 159.44, 158.46, 154.85 (C=NOH), 131.72, 128.80, 116.12, 99.24, 96.11, 94.64, 77.39, 55.67, 30.28. HR ESI-MS *m*/*z* calculated for C_16_H_15_NO_5_ [M + H]^+^ 302.1023, found [M + H]^+^ 302.1031.

*7,4*′*-Di-O-methylnaringenin oxime* (**2b**), Yield 80.8% (0.424 g), white powder, m.p. 155–157 °C. ^1^H-NMR (600 MHz, acetone-*d*_6_) δ (ppm): 11.03 (s, 1H, NOH), 10.41 (s, 1H, OH-5), 7.51–7.43 (m, 2H, AA′BB′, H-2′, H-6′), 7.03–6.96 (m, 2H, AA′BB′, H-3′, H-5′), 6.06 (d, *J* = 2.5 Hz, 1H, H-6), 6.05 (d, *J* = 2.5 Hz, 1H, H-8), 5.12 (dd, *J* = 11.9, 3.3 Hz, 1H, H-2), 3.82 (s, 3H, -CH_3_), 3.76 (s, 3H, -CH_3_), 3.48 (dd, *J* = 17.0, 3.3 Hz, 1H, H-3_a_), 2.81 (dd, *J* = 17.0, 11.9 Hz, 1H, H-3_b_). ^13^C-NMR (150 MHz, acetone-*d*_6_) δ (ppm): 163.49, 160.74, 160.65, 159.34, 154.73 (C=NOH), 132.85, 128.68, 114.72, 99.25, 96.14, 94.67, 77.22, 55.69, 55.59, 30.26. HR ESI-MS *m*/*z* calculated for C_17_H_17_NO_5_ [M + H]^+^ 316.1179, found [M + H]^+^ 316.1185.

*5,7,4*′*-Tri-O-methylnaringenin oxime* (**3b**), Yield 96.0% (0.302 g), white powder, m.p. 211–214 °C, lit. 200-202 °C [22]. ^1^H-NMR (600 MHz, DMSO-*d*_6_) δ (ppm): 11.05 (s, 1H, NOH), 7.42–7.37 (m, 2H, AA′BB′, H-2′, H-6′), 6.99–6.93 (m, 2H, AA′BB′, H-3′, H-5′), 6.24 (d, *J* = 2.3 Hz, 1H, H-6), 6.17 (d, *J* = 2.3 Hz, 1H, H-8), 5.03 (dd, *J* = 11.7, 3.3 Hz, 1H, H-2), 3.76 (s, 3H, -CH_3_), 3.75 (s, 3H, -CH_3_), 3.74 (s, 3H, -CH_3_), 3.33 (dd, *J* = 16.9, 3.3 Hz, 1H, H-3_a_), 2.69 (dd, *J* = 16.9, 11.7 Hz, 1H, H-3_b_). ^13^C-NMR (150 MHz, DMSO-*d*_6_) δ (ppm): 160.85, 159.21, 159.1, 158.6, 147.66 (C=NOH), 131.97, 127.82, 113.77, 101.88, 94.28, 93.24, 75.95, 55.66, 55.29, 55.12, 30.14. HR ESI-MS *m*/*z* calculated for C_18_H_19_NO_5_ [M + H]^+^ 330.1336, found [M + H]^+^ 330.1338, lit. HR ESI-MS *m*/*z* calculated for C_18_H_19_NO_5_ [M + H]^+^ 330.1341, found [M + H]^+^ 330.1335 [22].

*7-O-Ethylnaringenin oxime* (**4b**), Yield 96.9% (0.509 g), white powder, m.p. 203–205 °C. ^1^H-NMR (600 MHz, acetone-*d*_6_) δ (ppm): 11.01 (s, 1H, NOH), 10.38 (s, 1H, OH-5), 8.47 (s, 1H, OH-4′), 7.41–7.34 (m, 2H, AA′BB′, H-2′, H-6′), 6.92–6.85 (m, 2H, AA′BB′, H-3′, H-5′), 6.04 (d, *J* = 2.4 Hz, 1H, H-6), 6.02 (d, *J* = 2.4 Hz, 1H, H-8), 5.07 (dd, *J* = 12.0, 3.2 Hz, 1H, H-2), 4.02 (q, *J* = 7.0 Hz, 2H, -CH_2_-), 3.46 (dd, *J* = 17.1, 3.2 Hz, 1H, H-3_a_), 2.79 (dd, *J* = 17.1, 12.0 Hz, 1H, H-3_b_), 1.33 (t, *J* = 7.0 Hz, 3H, -CH_3_). ^13^C-NMR (150 MHz, acetone-*d*_6_) δ (ppm): 162.78, 160.63, 159.42, 158.46, 154.87 (C=NOH), 131.75, 128.80, 116.12, 99.15, 96.54, 95.07, 77.38, 64.16, 30.30, 14.97. HR ESI-MS *m*/*z* calculated for C_17_H_17_NO_5_ [M + H]^+^ 316.1179, found [M + H]^+^ 316.1191.

*7,4*′*-Di-O-ethylnaringenin oxime* (**5b**), Yield 83.8% (0.307 g), white powder, m.p. 160–162 °C. ^1^H-NMR (600 MHz, acetone-*d*_6_) δ (ppm): 11.01 (s, 1H, NOH), 10.38 (s, 1H, OH-5), 7.48–7.41 (m, 2H, AA′BB′, H-2′, H-6′), 7.00–6.91 (m, 2H, AA′BB′, H-3′, H-5′), 6.04 (d, *J* = 2.3 Hz, 1H, H-6), 6.03 (d, *J* = 2.3 Hz, 1H, H-8), 5.11 (dd, *J* = 11.9, 3.2 Hz, 1H, H-2), 4.07 (q, *J* = 7.0 Hz, 2H, -CH_2_-), 4.02 (q, *J* = 7.0 Hz, 2H, -CH_2_-), 3.47 (dd, *J* = 17.1, 3.2 Hz, 1H, H-3_a_), 2.81 (dd, *J* = 17.1, 11.9 Hz, 1H, H-3_b_), 1.37 (t, *J* = 7.0 Hz, 3H, -CH_3_), 1.34 (t, *J* = 7.0 Hz, 3H, -CH_3_). ^13^C-NMR (150 MHz, acetone-*d*_6_) δ (ppm): 162.80, 160.63, 160.06, 159.33, 154.76 (C=NOH), 132.73, 128.67, 115.23, 99.15, 96.57, 95.10, 77.22, 64.17, 64.05, 30.27, 15.10, 14.97. HR ESI-MS *m*/*z* calculated for C_19_H_21_NO_5_ [M + H]^+^ 344.1492, found [M + H]^+^ 344.1502.

*5,7,4*′*-Tri-O-ethylnaringenin oxime* (**6b**), Yield 95.5% (0.149 g), white powder, m.p. 169–171 °C. ^1^H-NMR (600 MHz, DMSO-*d*_6_) δ (ppm): 10.89 (s, 1H, NOH), 7.38 (d, *J* = 8.2 Hz, 2H, H-2′, H-6′), 6.93 (d, *J* = 8.2 Hz, 2H, H-3′, H-5′), 6.19 (s, 1H, H-6), 6.13 (s, 1H, H-8), 5.00 (d, *J* = 11.7 Hz, 1H, H-2), 4.11–3.99 (m, 6H, 3x-CH_2_-), 3.31 (d, *J* = 16.8 Hz, 1H, H-3_a_), 2.68 (dd, *J* = 16.8, 11.7 Hz, 1H, H-3_b_), 1.35–1.27 (m, 9H, 3x-CH_3_). ^13^C-NMR (150 MHz, DMSO-*d*_6_) δ (ppm): 159.99, 158.70, 158.36, 158.30, 147.78 (C=NOH), 131.88, 127.81, 114.20, 102.26, 94.68, 94.64, 76.01, 63.87, 63.16, 63.02, 30.33, 14.63, 14.60, 14.53. HR ESI-MS *m*/*z* calculated for C_21_H_25_NO_5_ [M + H]^+^ 372.1805, found [M + H]^+^ 372.1812.

*7-O-Pentylnaringenin oxime* (**7b**), Yield 86.2% (0.450 g), white powder, m.p. 189–191 °C. ^1^H-NMR (600 MHz, acetone-*d*_6_) δ (ppm): 11.01 (s, 1H, NOH), 10.37 (s, 1H, OH-5), 8.46 (s, 1H, OH-4’), 7.42–7.34 (m, 2H, AA′BB′, H-2′, H-6′), 6.93–6.86 (m, 2H, AA′BB′, H-3′, H-5′), 6.07 (d, *J* = 2.5 Hz, 1H, H-6), 6.06 (d, *J* = 2.5 Hz, 1H, H-8), 5.07 (dd, *J* = 12.0, 3.2 Hz, 1H, H-2), 3.96 (t, *J* = 6.6 Hz, 2H, -CH_2_-), 3.46 (dd, *J* = 17.1, 3.2 Hz, 1H, H-3_a_), 2.79 (dd, *J* = 17.1, 12.0 Hz, 1H, H-3_b_), 1.74 (p, *J* = 6.6 Hz, 2H, -CH_2_-), 1.47–1.33 (m, 4H, 2x-CH_2_-), 0.92 (t, *J* = 7.2 Hz, 3H, -CH_3_). ^13^C-NMR (150 MHz, acetone-*d*_6_) δ (ppm): 162.95, 160.63, 159.42, 158.45, 154.87 (C=NOH), 131.76, 128.79, 116.12, 99.13, 96.58, 95.11, 77.37, 68.64, 30.30, 30.06, 28.89, 23.07, 14.29. HR ESI-MS *m*/*z* calculated for C_20_H_23_NO_5_ [M + H]^+^ 358.1649, found [M + H]^+^ 358.1651.

*7,4*′*-Di-O-pentylnaringenin oxime* (**8b**), Yield 99.5% (0.474 g), white powder, m.p. 71–74 °C. ^1^H-NMR (600 MHz, acetone-*d*_6_) δ (ppm): 11.00 (s, 1H, NOH), 10.38 (s, 1H, OH-5), 7.49–7.42 (m, 2H, AA′BB′, H-2′, H-6′), 7.01–6.95 (m, 2H, AA′BB′, H-3′, H-5′), 6.08 (d, *J* = 2.4 Hz, 1H, H-6), 6.06 (d, *J* = 2.4 Hz, 1H, H-8), 5.11 (dd, *J* = 11.9, 3.2 Hz, 1H, H-2), 4.02 (t, *J* = 6.5 Hz, 2H, -CH_2_-), 3.96 (t, *J* = 6.5 Hz, 2H, -CH_2_-), 3.47 (dd, *J* = 17.1, 3.2 Hz, 1H, H-3_a_), 2.81 (dd, *J* = 17.1, 11.9 Hz, 1H, H-3_b_), 1.82–1.70 (m, 4H, 2x-CH_2_-), 1.48–1.36 (m, 8H, 4x-CH_2_-), 0.93 (t, *J* = 7.4 Hz, 3H, -CH_3_), 0.92 (t, *J* = 7.4 Hz, 3H, -CH_3_). ^13^C-NMR (150 MHz, acetone-*d*_6_) δ (ppm): 162.08, 159.75, 159.35, 158.44, 153.88 (C=NOH), 131.84, 127.78, 114.38, 98.26, 95.72, 94.25, 76.34, 67.77, 67.70, 29.39, 29.05, 28.93, 28.08, 28.01, 22.24, 22.19, 13.43, 13.41. HR ESI-MS *m*/*z* calculated for C_25_H_33_NO_5_ [M + H]^+^ 428.2432, found [M + H]^+^ 428.2436.

*7-O-Dodecylnaringenin oxime* (**9b**), Yield 95.2% (0.197 g), white powder, m.p. 156–159 °C. ^1^H-NMR (600 MHz, acetone-*d*_6_) δ (ppm): 11.01 (s, 1H, NOH), 10.37 (s, 1H, OH-5), 8.47 (s, 1H, OH-4′), 7.41–7.34 (m, 2H, AA′BB′, H-2′, H-6′), 6.92–6.86 (m, 2H, AA′BB′, H-3′, H-5′), 6.05 (d, *J* = 2.5 Hz, 1H, H-6), 6.03 (d, *J* = 2.5 Hz, 1H, H-8), 5.07 (dd, *J* = 12.0, 3.1 Hz, 1H, H-2), 3.97 (t, *J* = 6.6 Hz, 2H, -CH_2_-), 3.46 (dd, *J* = 17.1, 3.1 Hz, 1H, H-3_a_), 2.79 (dd, *J* = 17.1, 12.0 Hz, 1H, H-3_b_), 1.74 (p, *J* = 6.7 Hz, 2H, -CH_2_-), 1.49–1.41 (m, 2H, -CH_2_-), 1.38–1.22 (m, 16H, 8x-CH_2_-), 0.87 (t, *J* = 7.0 Hz, 3H, -CH_3_). ^13^C-NMR (150 MHz, acetone-*d*_6_) δ (ppm): 162.95, 160.62, 159.41, 158.45, 154.87 (C=NOH), 131.76, 128.79, 116.11, 99.12, 96.58, 95.11, 77.37, 68.65, 32.63, 30.38, 30.36, 30.31, 30.07, 30.05, 26.70, 23.33, 14.36. HR ESI-MS *m*/*z* calculated for C_27_H_37_NO_5_ [M + H]^+^ 456.2745, found [M + H]^+^ 456.2767.

*7,4*′*-Di-O-dodecylnaringenin oxime* (**10b**), Yield 86.7% (0.107 g), white powder, m.p. 82–85 °C. ^1^H-NMR (600 MHz, acetone-*d*_6_) δ (ppm): 11.01 (s, 1H, NOH), 10.39 (s, 1H, OH-5), 7.48–7.43 (m, 2H, AA′BB′ H-2′, H-6′), 7.01–6.95 (m, 2H, AA′BB′, H-3′, H-5′), 6.05 (d, *J* = 2.4 Hz, 1H, H-6), 6.04 (d, *J* = 2.4 Hz, 1H, H-8), 5.11 (dd, *J* = 11.8, 3.2 Hz, 1H, H-2), 4.03 (t, *J* = 6.6 Hz, 2H, -CH_2_-), 3.97 (t, *J* = 6.6 Hz, 2H, -CH_2_-), 3.47 (dd, *J* = 17.0, 3.2 Hz, 1H, H-3_a_), 2.79 (dd, *J* = 17.0, 11.8 Hz, 1H, H-3_b_), 1.82–1.71 (m, 4H, 2x-CH_2_-), 1.53–1.42 (m, 4H, 2x-CH_2_-), 1.40–1.26 (m, 32H, 16x-CH_2_-), 0.88 (t, *J* = 7.0 Hz, 3H, -CH_3_), 0.88 (t, *J* = 7.0 Hz, 3H, -CH_3_). ^13^C-NMR (150 MHz, acetone-*d*_6_) δ (ppm): 162.96, 160.63, 160.23, 159.32, 154.75 (C=NOH), 132.71, 128.65, 115.26, 99.13, 96.61, 95.13, 77.22, 68.66, 68.58, 32.64, 30.38, 30.37, 30.35, 30.33, 30.32, 30.28, 30.06, 26.78, 26.70, 23.34, 14.37. HR ESI-MS *m*/*z* calculated for C_39_H_61_NO_5_ [M + H]^+^ 624.4623, found [M + H]^+^ 624.4613.

### 3.5. Biological Activity

Antimicrobial activity was performed on two strains of bacteria: *E. coli* ATCC10536 and *S. aureus* DSM799 and four strains of fungi: *C. albicans* DSM1386, *F. linii* KB-F1, *A. alternata* CBS1526 and *A. niger* DSM1957. All the microorganisms were from the collection of the Faculty of Biotechnology and Food Microbiology, Wroclaw University of Environmental and Life Sciences. The culture medium for bacteria was nutrient broth (Biocorp, Warsaw, Poland), and that for fungi was YM medium, which consisted of 3 g yeast extract, 3 g malt extract, 5 g bacteriological peptone and 10 g of glucose dissolved in 1 L of distilled water. Tests were prepared on 100-well microtiter plates, with the working volume in each well being 300 μL: 280 μL of culture medium, 10 μL of microorganism suspension and 10 μL of naringenin derivative dissolved in dimethyl sulfoxide (0.3% (*w*/*v*)). The final concentration of the derivative was 0.1% (*w*/*v*). Each culture was carried out in 3 replications. The optical density of the cell suspension was measured on Bioscreen C (Automated Growth Curve Analysis System Lab System, Helsinki, Finland) at 560 nm automatically, at regular intervals of 30 min for 2–3 days. Cell cultures were maintained at 28 °C on a continuous shaker. To prepare the growth curves for each strain, the mean values of the absorbance of the medium as a function of time were used. The resulting antimicrobial activity was expressed as the increase in optical density (ΔOD) and was compared to that of the control cultures in the medium supplemented with dimethyl sulfoxide.

## 4. Conclusions

In this paper, we report the synthesis and evaluation of the antimicrobial activity of the *O*-alkyl derivatives of naringenin and their oximes including novel compounds **7a**–**10a**, **2b,** and **4b**–**10b**.

The highest inhibitory effect against *E. coli* ATCC10536, *A. alternata* CBS1526, *F. linii* KB-F1, and *A. niger* DSM1957 was observed for the novel 7,4′-di-*O*-pentylnaringenin (**8a**). Moreover, 7-*O*-dodecylnaringenin (**9a**) prevented the growth of *E. coli* ATCC10536. Furthermore, compound **10a**, which has one more dodecyl group attached at position C-4′, presented the same activity against *F. linii* KB-F1. Introduction of the oxime group afforded 8 new derivatives, which were never described in the literature. The best inhibitory effect was observed for the novel 7-*O*-dodecylnaringenin oxime (**9b**). Our results showed that elongation of the *O*-alkyl groups attached to positions C-7 and C-4′ in naringenin increases the antimicrobial activity. Moreover, replacement of carbonyl with the oxime group enhanced the inhibitory effect, especially the antifungal activity.

## Figures and Tables

**Table 1:** Antimicrobial activity of *O*-alkyl derivatives of naringenin **1a**–**10a**.

Strain		*E. coli*	*S. aureus*	*C. albicans*	*A. alternata*	*F. linii*	*A. niger*
Control	Lag-phase (h)	4.0	2.5	3.0	16.5	14.5	11.0
	∆OD	1.65	1.74	1.60	1.87	1.96	2.14
**NG**	Lag-phase (h)	15.0	4.5	5.0	20.0	-	5.5
	∆OD	1.30	1.49	1.50	1.34	0	1.74
**1a**	Lag-phase (h)	5.5	3.5	5.0	21.5	26.0	9.0
	∆OD	0.75	1.59	1.45	0.72	1.17	1.55
**2a**	Lag-phase (h)	4.0	4.0	4.0	16.0	9.5	12.5
	∆OD	0.63	1.73	1.09	0.49	0.72	1.49
**3a**	Lag-phase (h)	4.5	4.0	5.0	14.0	19.5	15.0
	∆OD	0.53	1.73	1.57	1.31	1.81	1.21
**4a**	Lag-phase (h)	4.0	2.5	5.5	25.0	26.5	6.5
	∆OD	0.52	1.65	1.35	0.46	1.18	1.36
**5a**	Lag-phase (h)	4.5	5.0	5.5	18.0	24.0	7.5
	∆OD	0.51	1.64	0.93	0.48	0.88	1.03
**6a**	Lag-phase (h)	5.0	2.5	5.5	19.5	14.0	13.0
	∆OD	0.24	1.07	0.50	0.47	0.52	0.54
**7a**	Lag-phase (h)	4.0	4.0	7.0	19.0	25.5	32.5
	∆OD	0.51	1.67	1.19	1.00	1.44	1.00
**8a**	Lag-phase (h)	-	4.5	6.5	9.5	34.0	-
	∆OD	0	1.46	1.00	0.47	0.33	0
**9a**	Lag-phase (h)	-	26.0	5.5	23.0	3.5	38.5
	∆OD	0	0.83	0.97	0.42	0.98	0.96
**10a**	Lag-phase (h)	3.0	3.0	0.5	33.0	-	6.0
	∆OD	0.23	0.91	1.19	1.63	0	1.58

NG—naringenin; OD—Optical Density (OD was measured for λ 560 nm).

**Table 2:** Antimicrobial activity of oximes **1b**–**10b**.

Strain		*E. coli*	*S. aureus*	*C. albicans*	*A. alternata*	*F. linii*	*A. niger*
Control	Lag-phase (h)	4.0	2.5	3.0	16.5	14.5	11.0
	∆OD	1.65	1.74	1.60	1.87	1.96	2.14
**NG-OX**	Lag-phase (h)	-	3.5	4.0	21.5	29.0	-
	∆OD	0	1.66	1.69	0.96	1.20	0
**1b**	Lag-phase (h)	5.0	4.0	5.0	37.5	26.5	45.5
	∆OD	0.73	1.46	1.30	0.69	1.41	0.49
**2b**	Lag-phase (h)	3.5	2.0	4.0	16.0	13.0	4.0
	∆OD	0.82	1.46	0.68	0.77	0.62	0.59
**3b**	Lag-phase (h)	4.0	2.5	10.0	11.5	11.0	10.0
	∆OD	0.74	1.90	1.29	1.10	1.51	1.10
**4b**	Lag-phase (h)	4.0	2.5	3.0	16.0	12.0	11.0
	∆OD	0.80	1.20	0.81	1.03	0.92	0.88
**5b**	Lag-phase (h)	4.5	2.0	4.0	19.0	13.0	5.0
	∆OD	0.29	0.95	0.41	0.51	0.55	0.40
**6b**	Lag-phase (h)	6.0	4.5	11.0	11.5	10.5	9.5
	∆OD	0.30	1.29	1.30	0.69	1.47	0.76
**7b**	Lag-phase (h)	5.0	2.0	3.0	16.0	13.0	11.0
	∆OD	0.45	0.97	0.77	1.02	0.82	0.83
**8b**	Lag-phase (h)	-	3.5	6.0	18.5	22.0	37.5
	∆OD	0	1.27	0.87	1.24	0.53	1.05
**9b**	Lag-phase (h)	-	4.5	3.0	34.5	-	10.5
	∆OD	0	0.35	0.22	0.54	0	0.58
**10b**	Lag-phase (h)	0.5	1.0	1.0	31.5	-	7.0
	∆OD	0.27	0.80	1.03	1.24	0	1.71

NG-OX—naringenin oxime; OD—Optical Density (OD was measured for λ 560 nm).

## Supplementary Materials

The following are available online.
