# Supplementary material for: Synthesis and Biological Activity of Novel O-Alkyl Derivatives of Naringenin and Their Oximes
Source: Molecules. 2017 Sep 6;22(9):1485. doi: 10.3390/molecules22091485 (PMC6151618; doi:10.3390/molecules22091485)
Supplement: Supplementary file 1 [file molecules-22-01485-s001.pdf]

Synthesis and Biological Activity of Novel *O*-alkyl Derivatives of Naringenin and Their Oximes

Joanna Kozłowska, Bartłomiej Potaniec, Barbara Żarowska and Mirosław Anioł

Supplementary materials

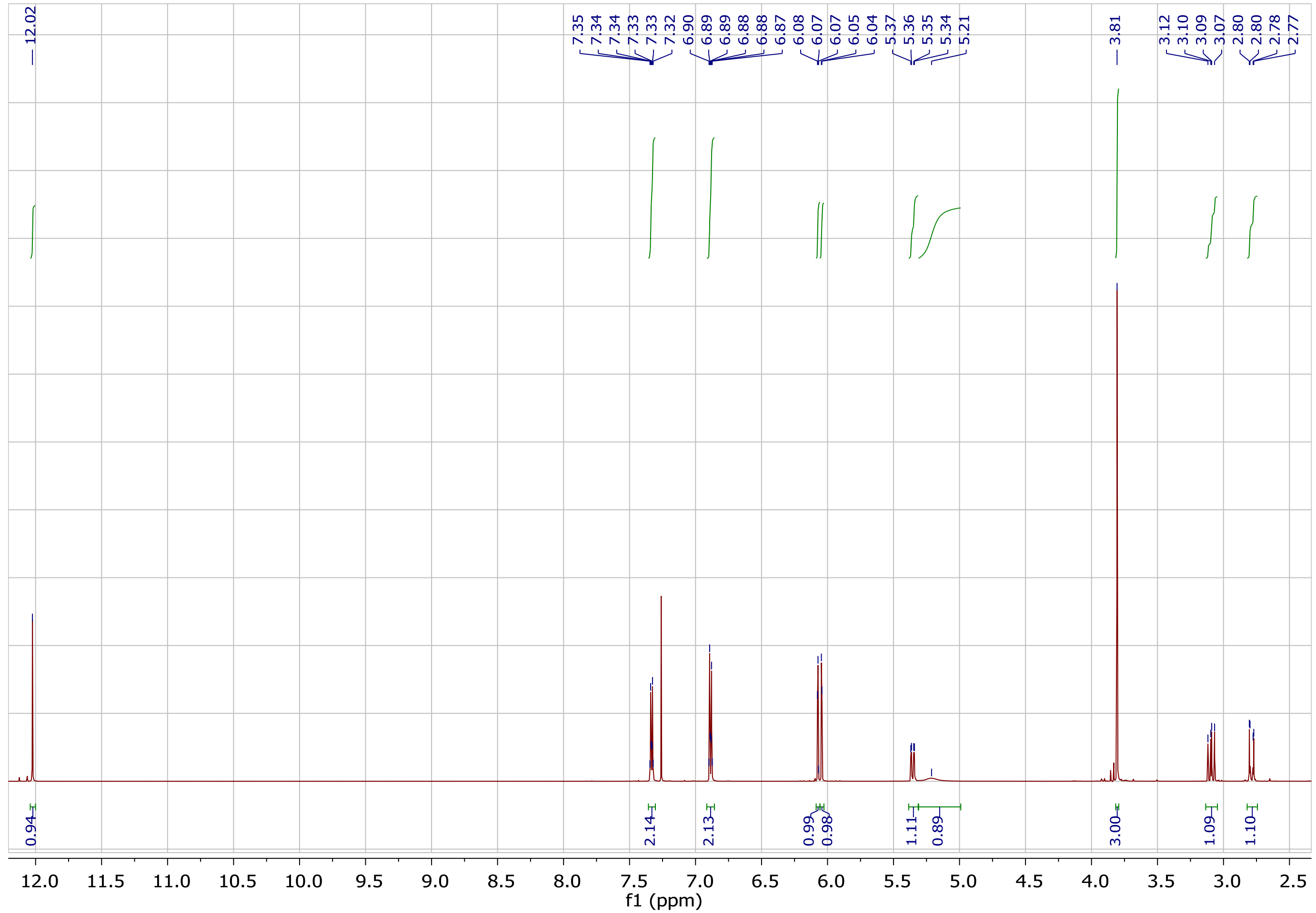

Figure S1. ^{1}H-NMR (600 MHz, $CDCl_3$) spectrum of compound **1a**

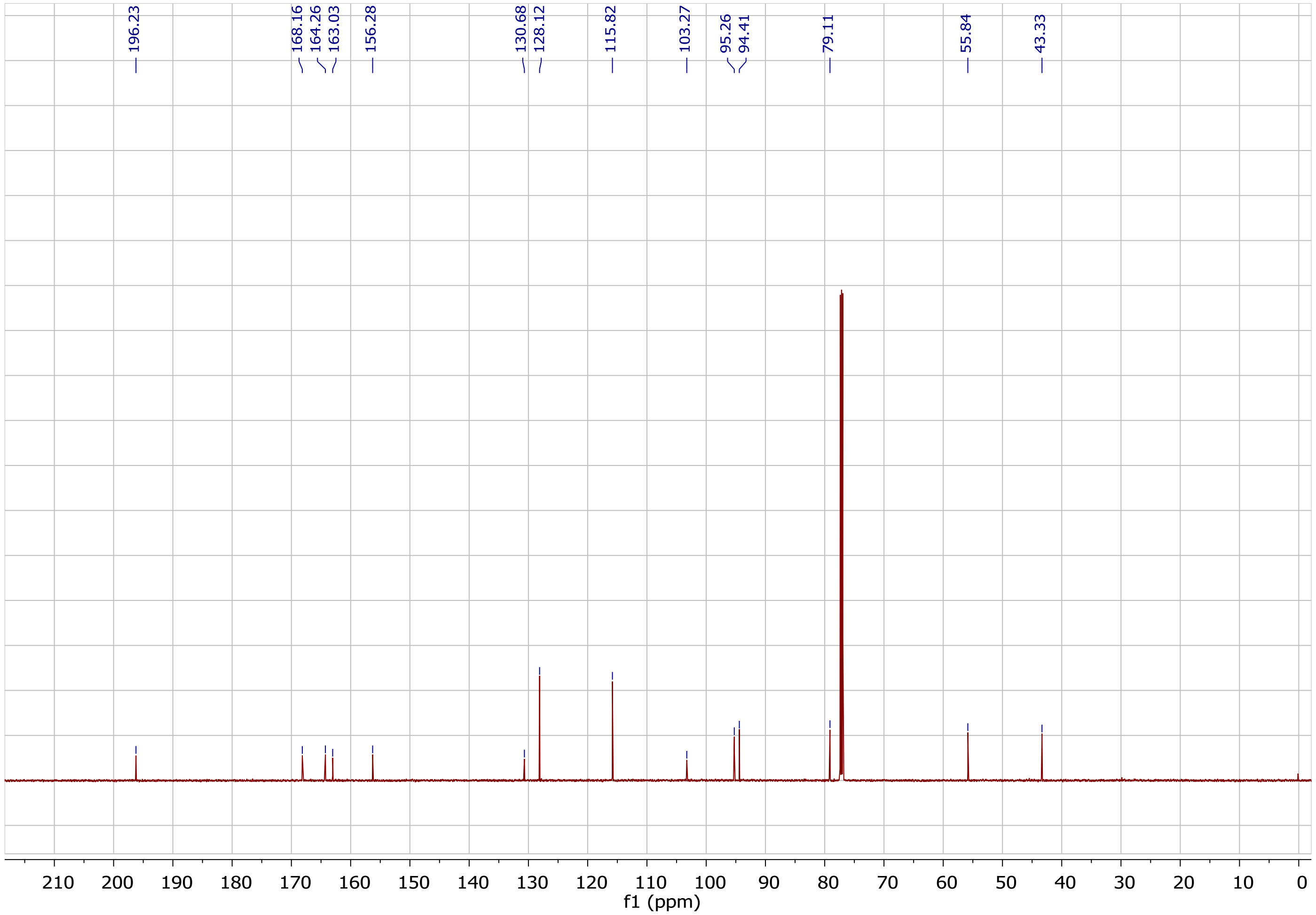

Figure S2. ^{13}C-NMR (150 MHz, $CDCl_3$) spectrum of compound **1a**

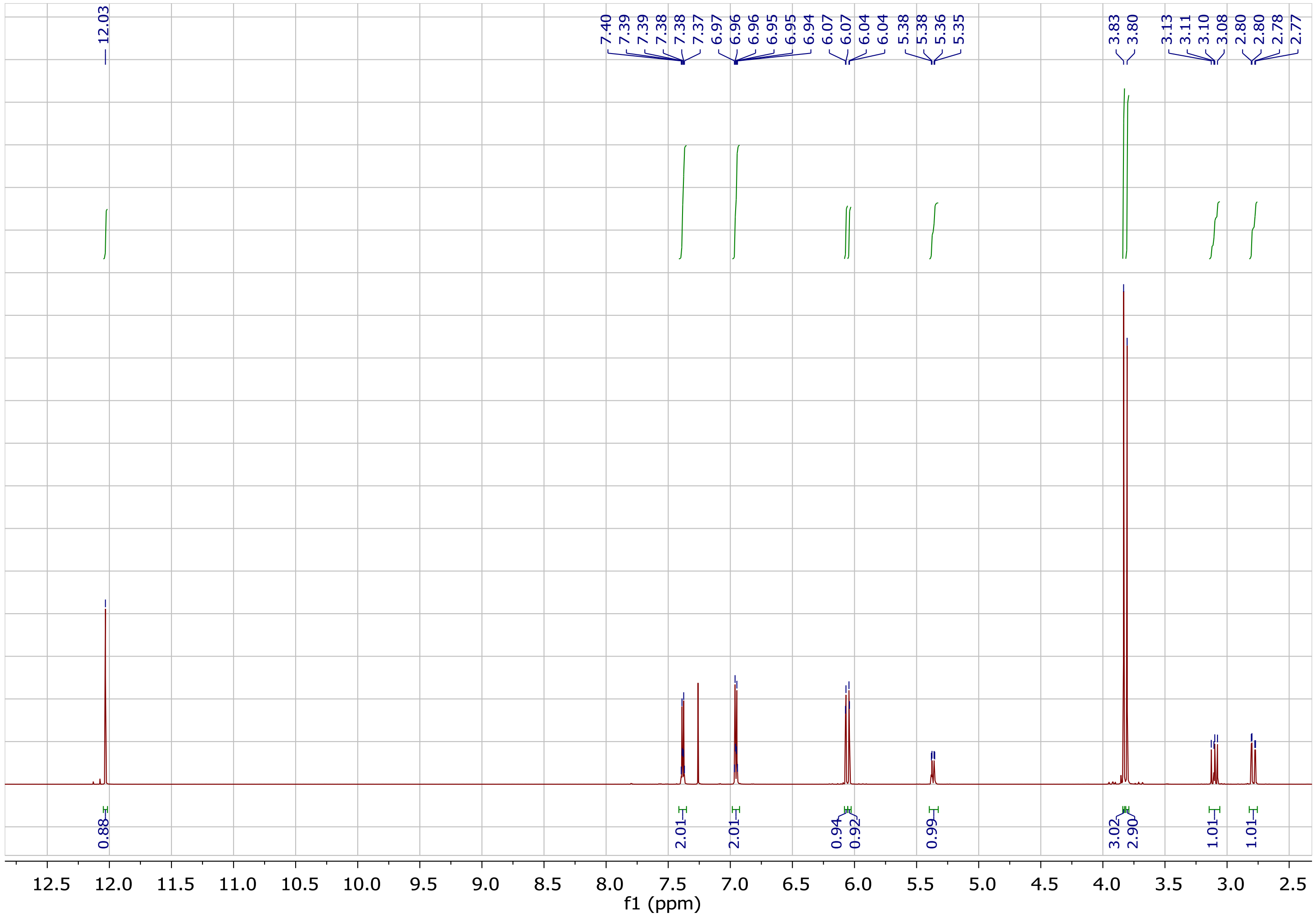

Figure S3. ^{1}H-NMR (600 MHz, $CDCl_3$) spectrum of compound **2a**

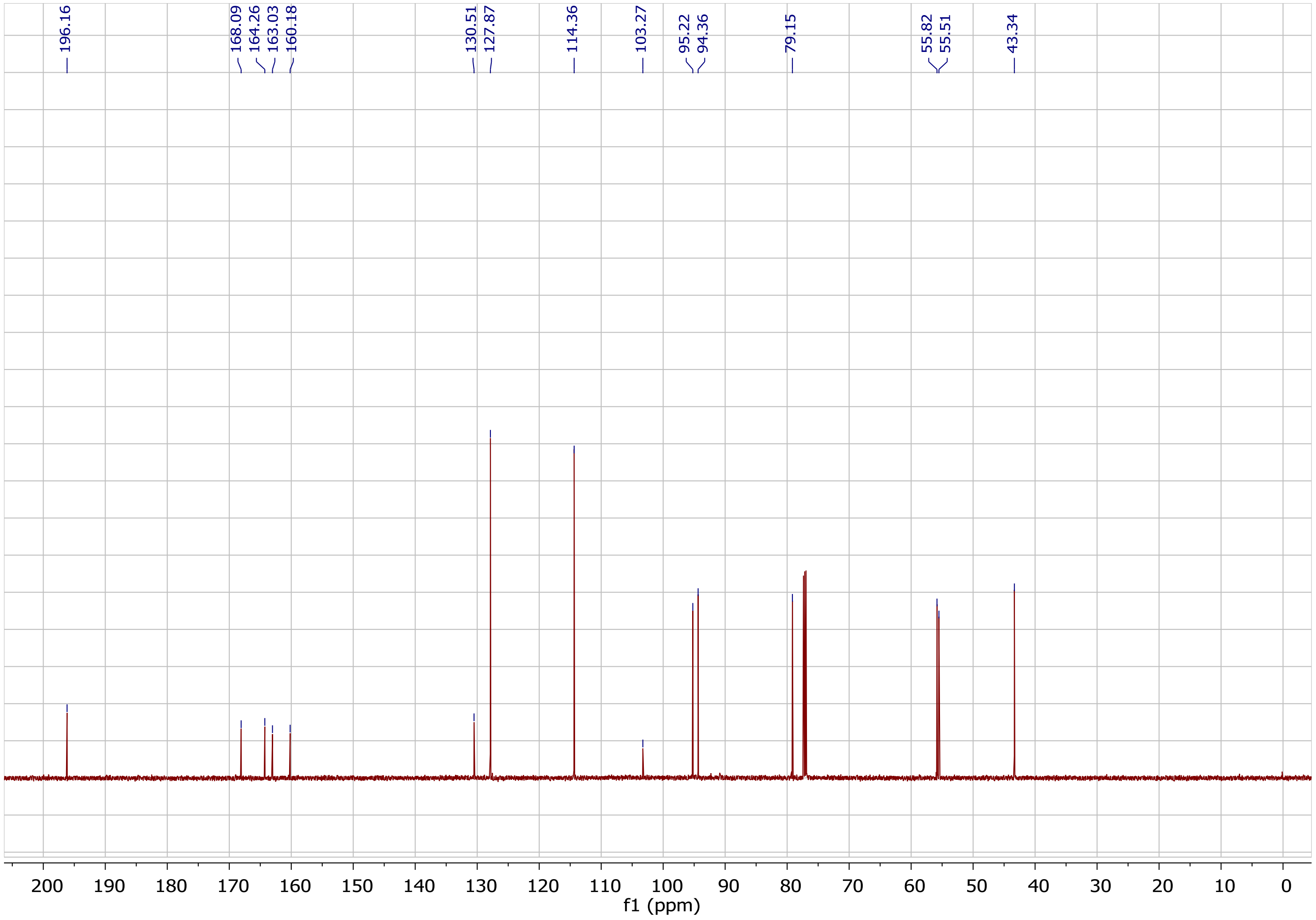

Figure S4. ^{13}C-NMR (150 MHz, $CDCl_3$) spectrum of compound **2a**

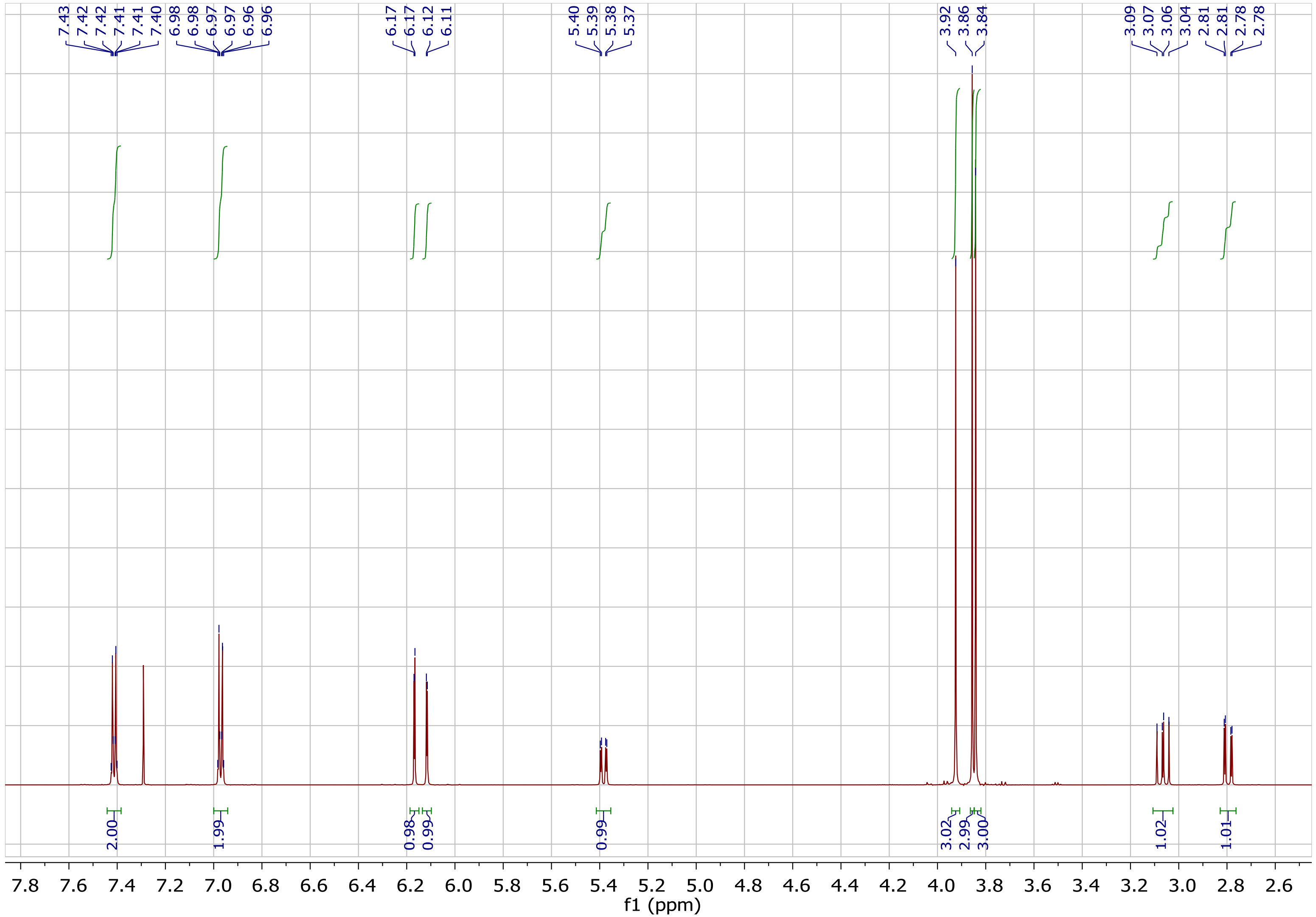

Figure S5. ^{1}H-NMR (600 MHz, $CDCl_3$) spectrum of compound **3a**

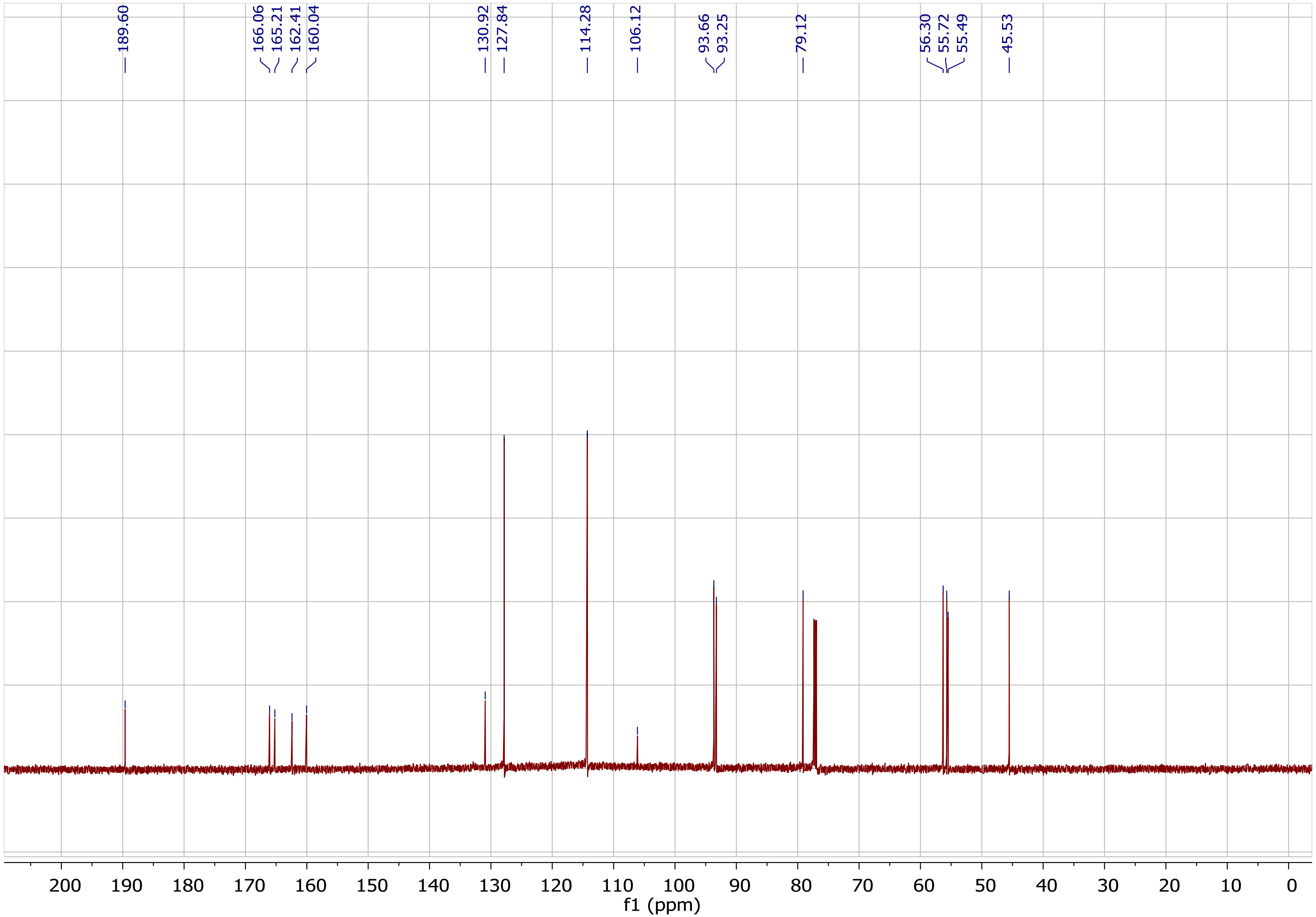

Figure S6. ^{13}C-NMR (150 MHz, $CDCl_3$) spectrum of compound **3a**

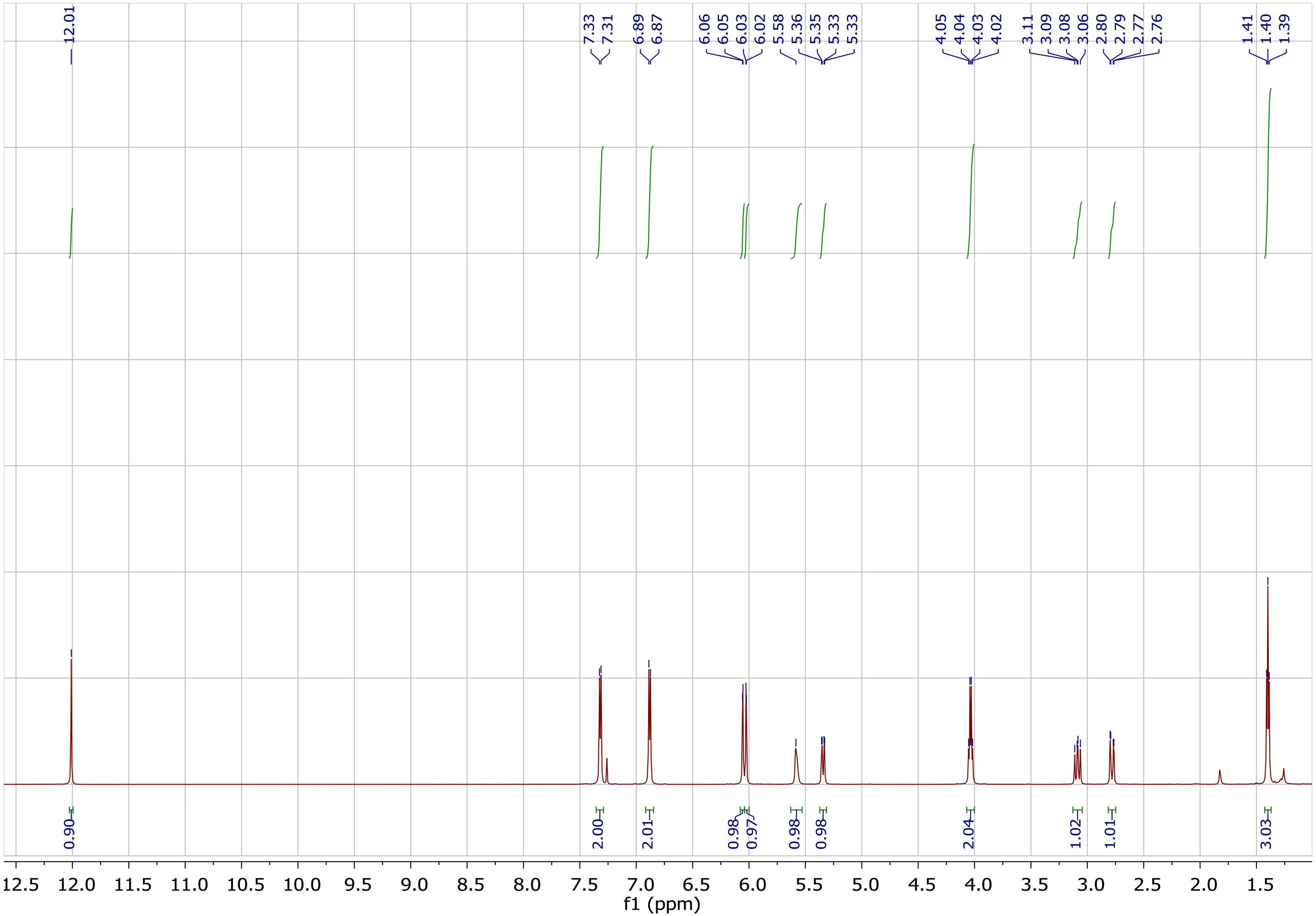

Figure S7. ^{1}H-NMR (600 MHz, $CDCl_3$) spectrum of compound **4a**

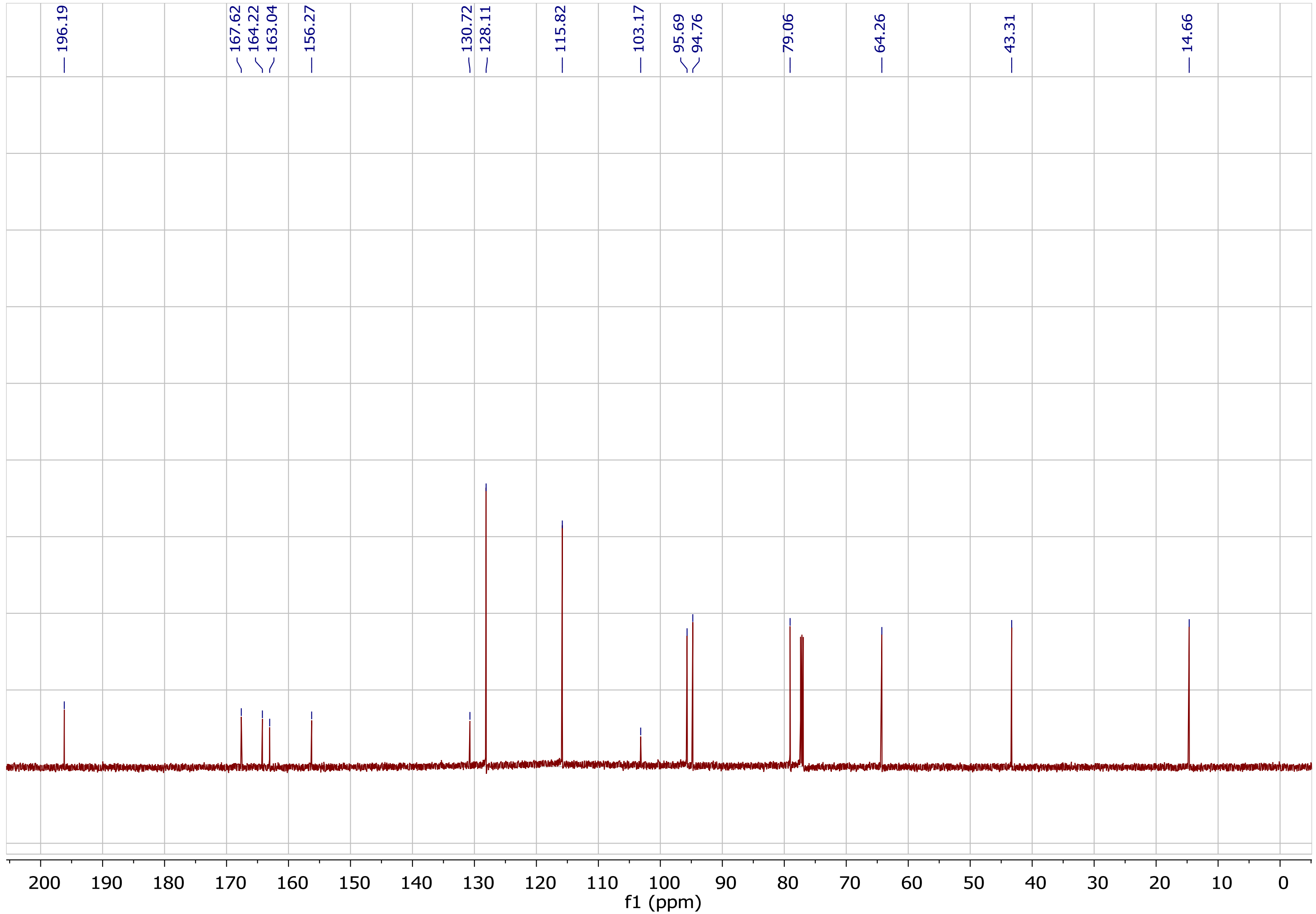

Figure S8. ^{13}C-NMR (150 MHz, $CDCl_3$) spectrum of compound **4a**

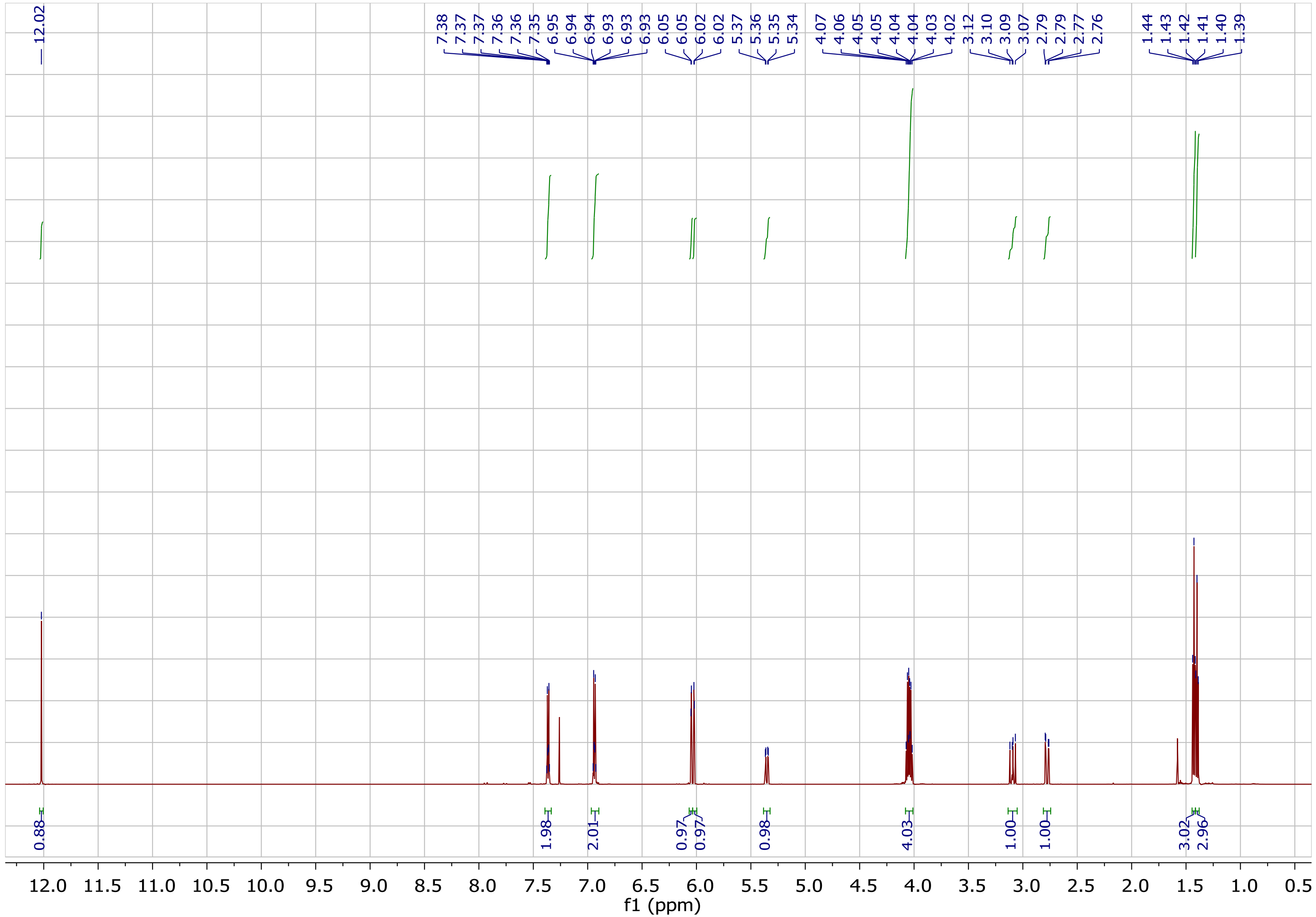

Figure S9. ^{1}H-NMR (600 MHz, $CDCl_3$) spectrum of compound **5a**

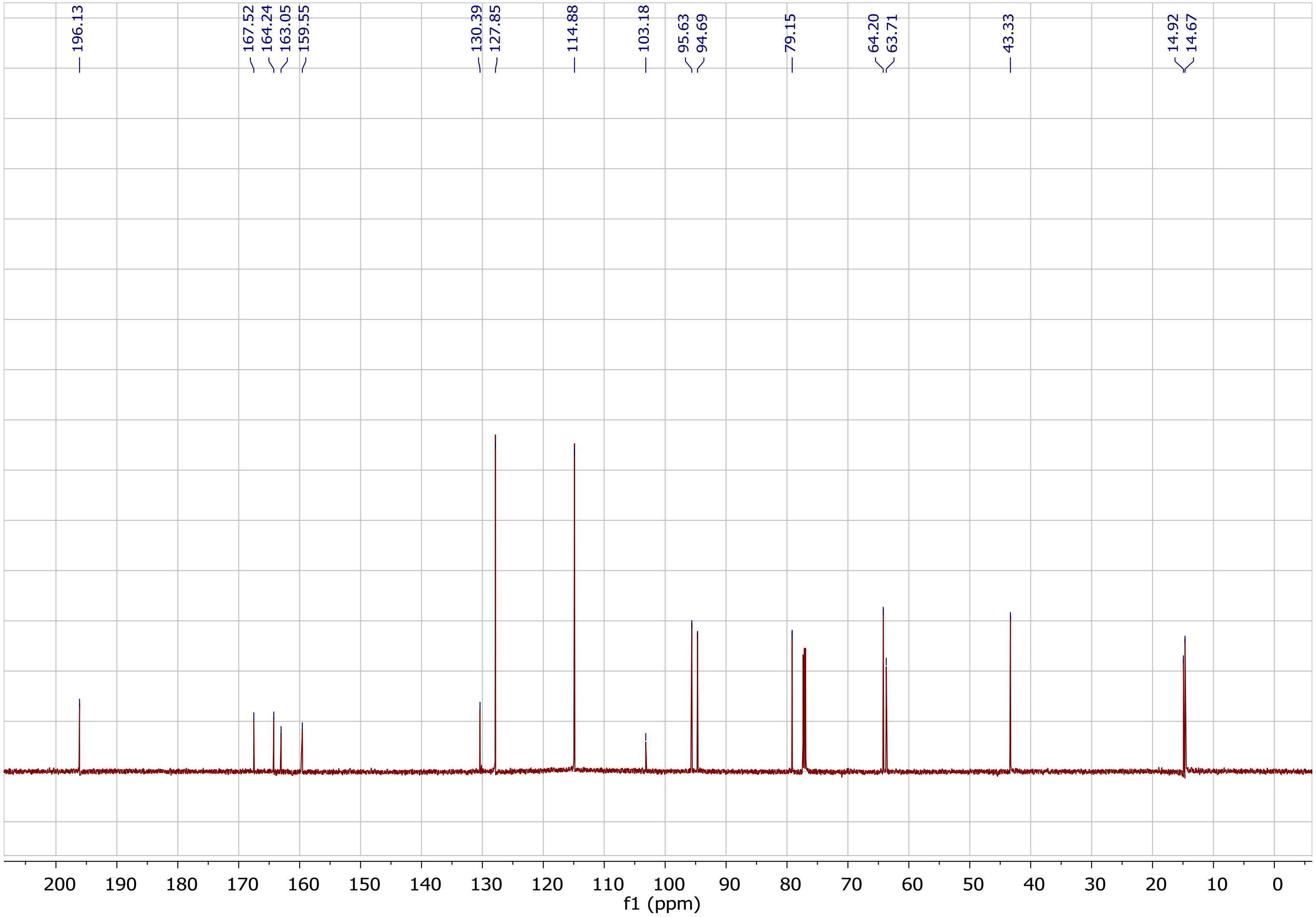

Figure S10. ^{13}C-NMR (150 MHz, $CDCl_3$) spectrum of compound **5a**

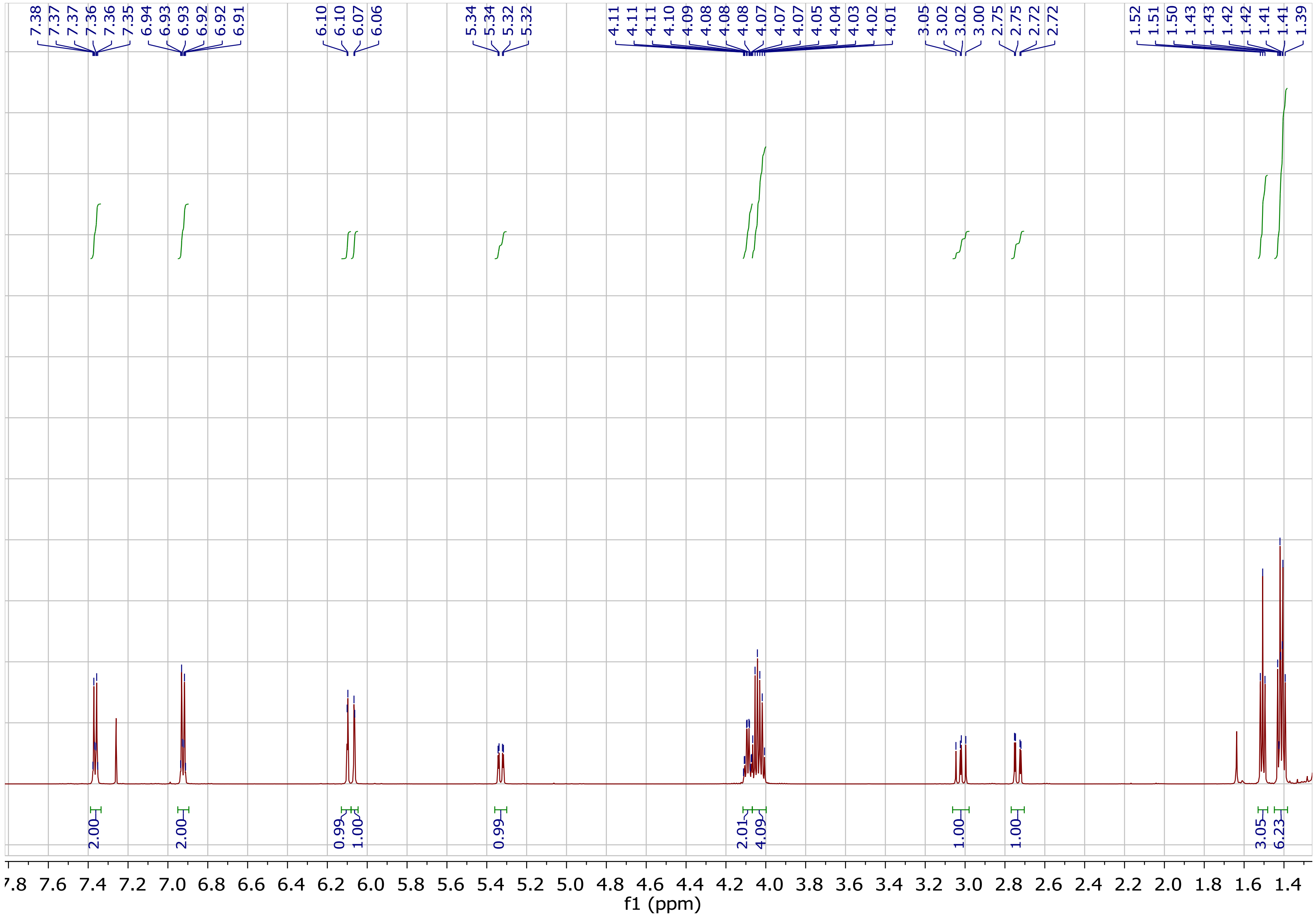

Figure S11. ^{1}H-NMR (600 MHz, $CDCl_3$) spectrum of compound **6a**

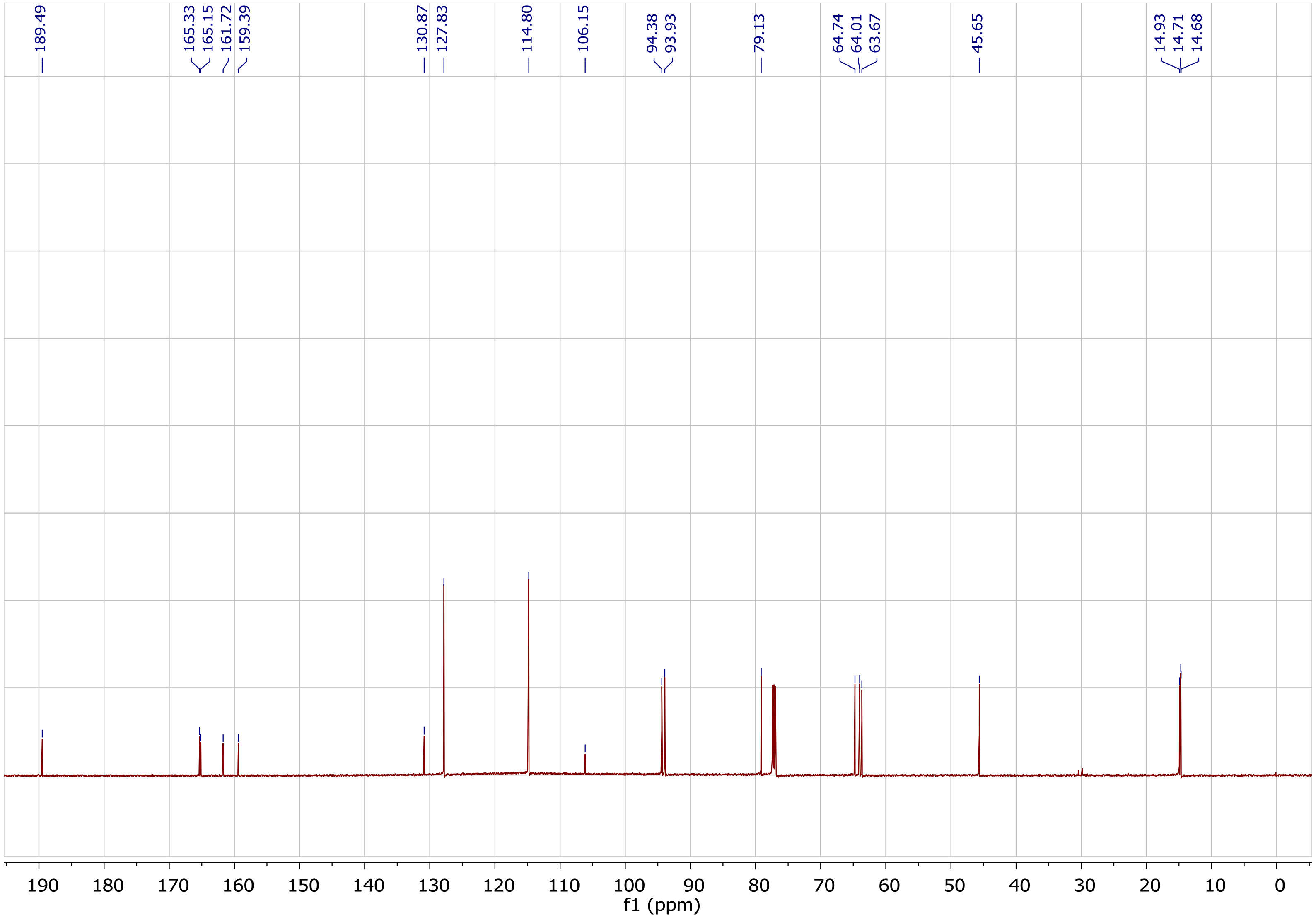

Figure S12. ^{13}C-NMR (150 MHz, $CDCl_3$) spectrum of compound **6a**

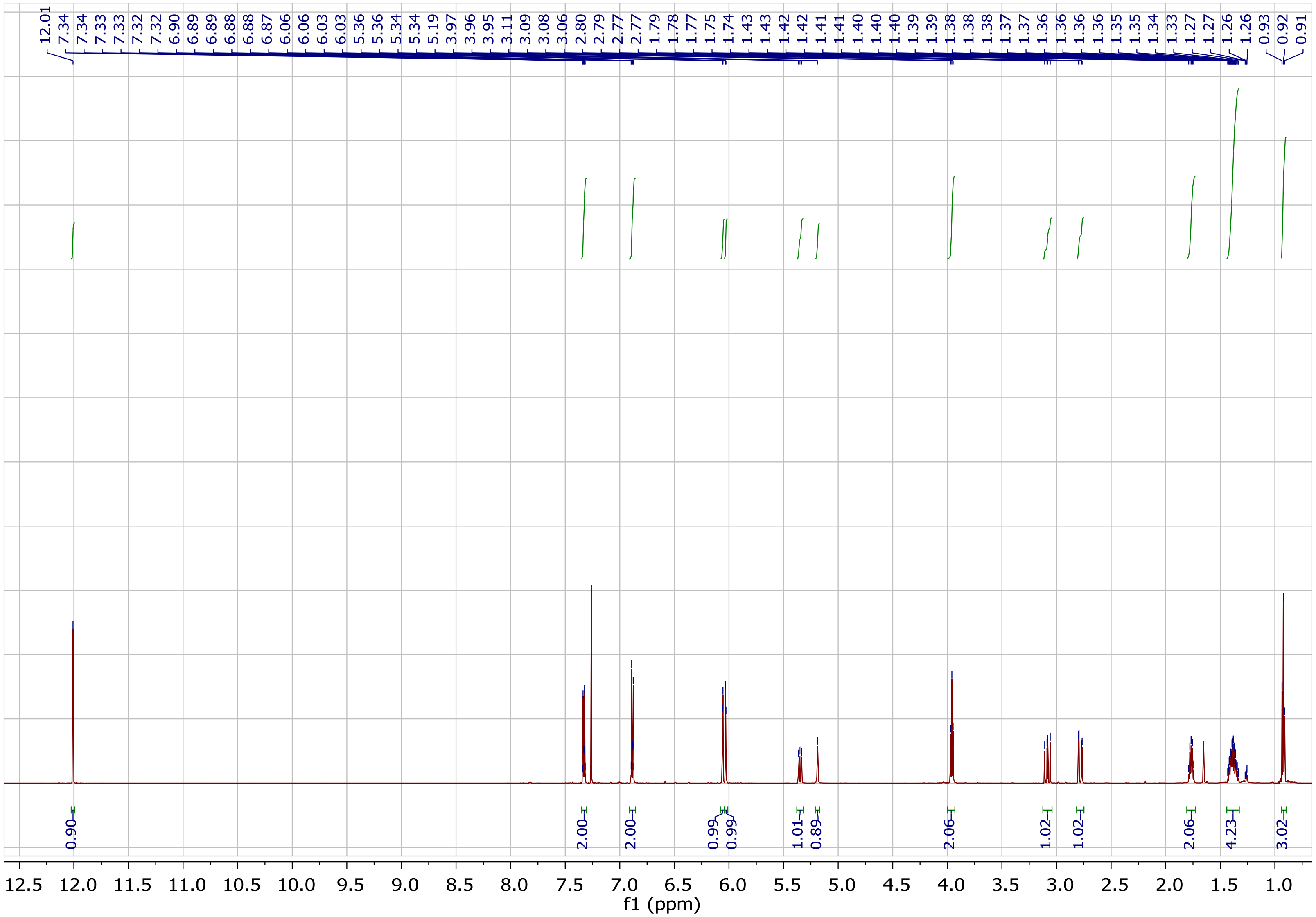

Figure S13. ^{1}H-NMR (600 MHz, $CDCl_3$) spectrum of compound **7a**

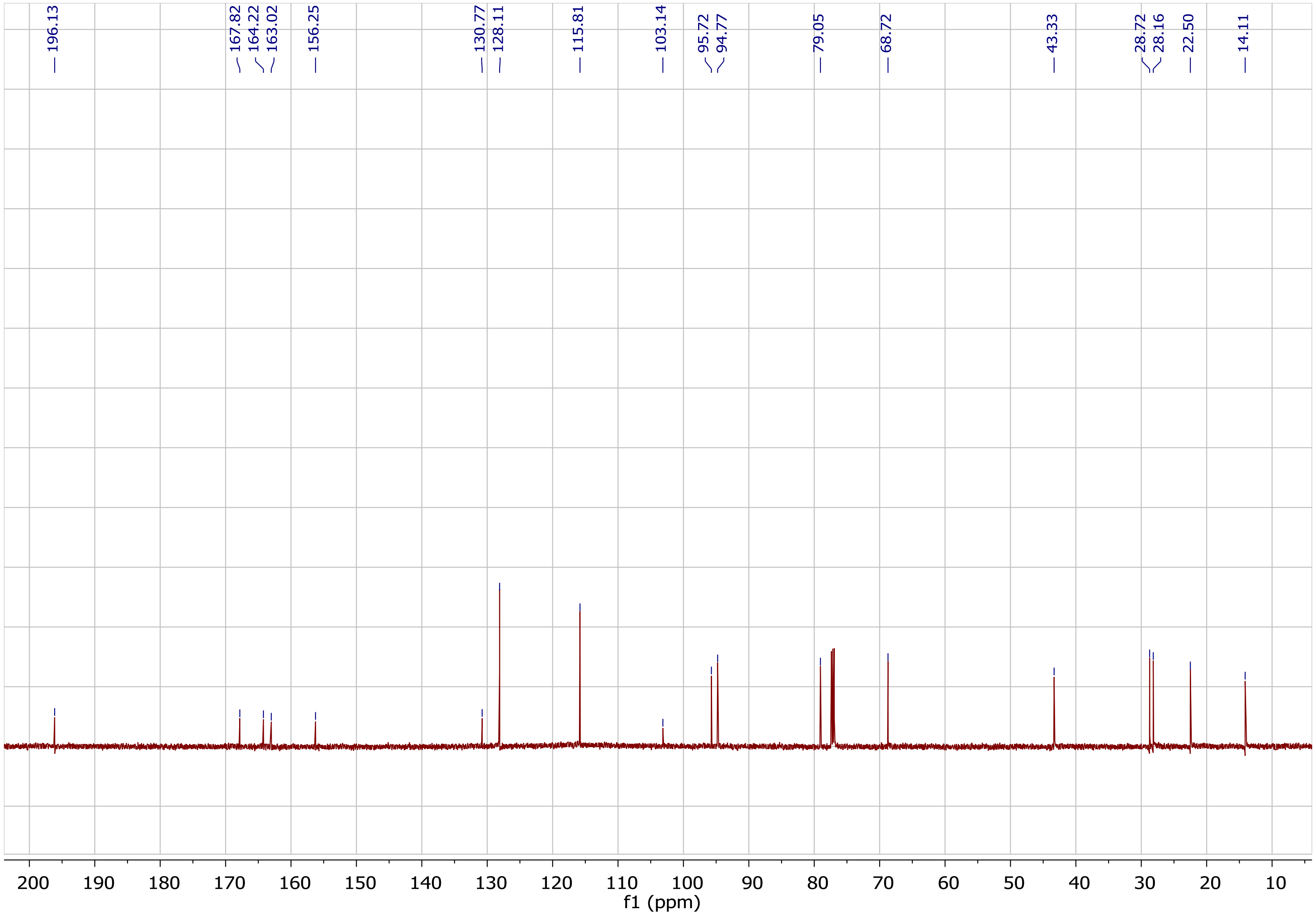

Figure S14. ^{13}C-NMR (150 MHz, $CDCl_3$) spectrum of compound **7a**

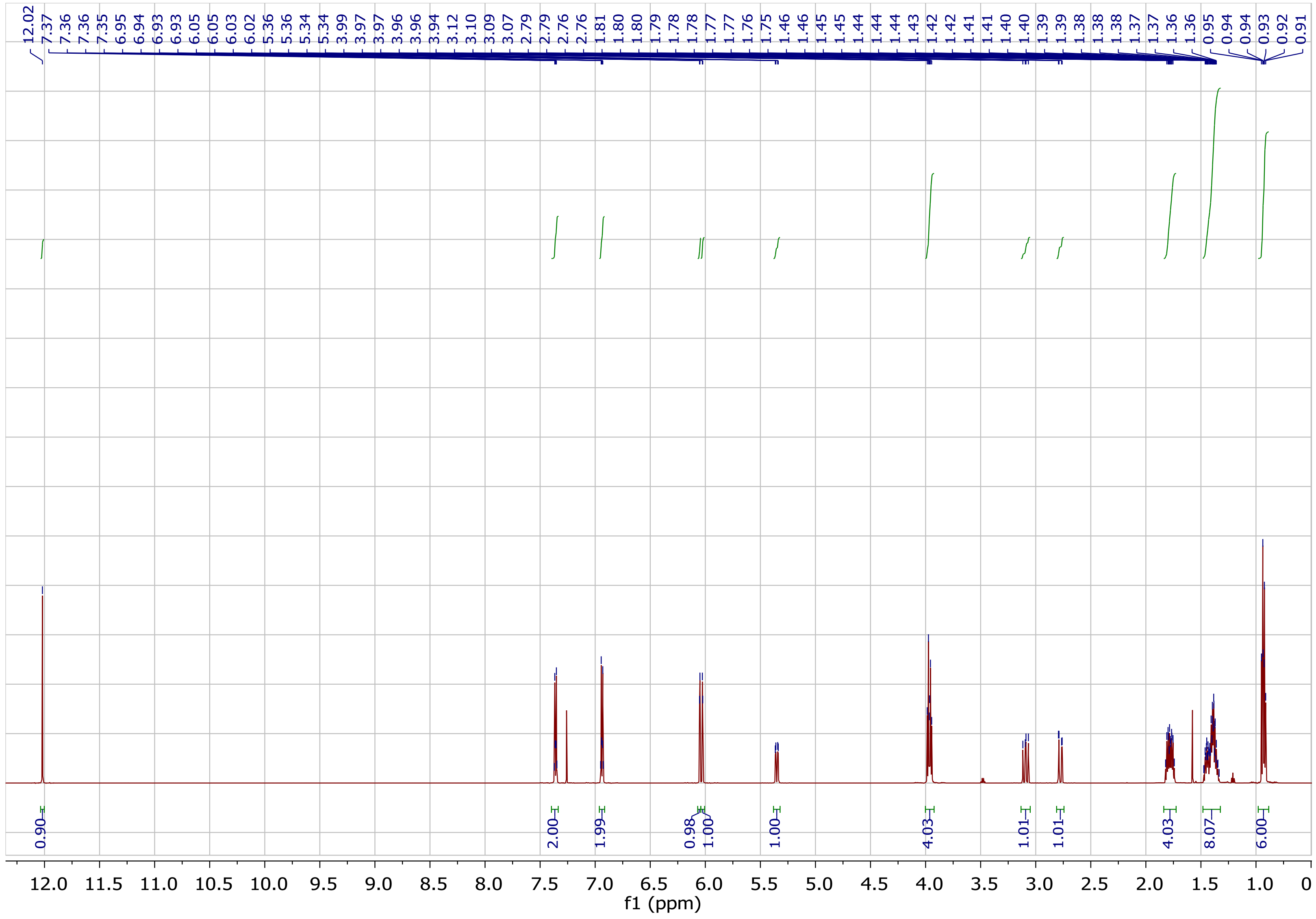

Figure S15. ^{1}H-NMR (600 MHz, $CDCl_3$) spectrum of compound **8a**

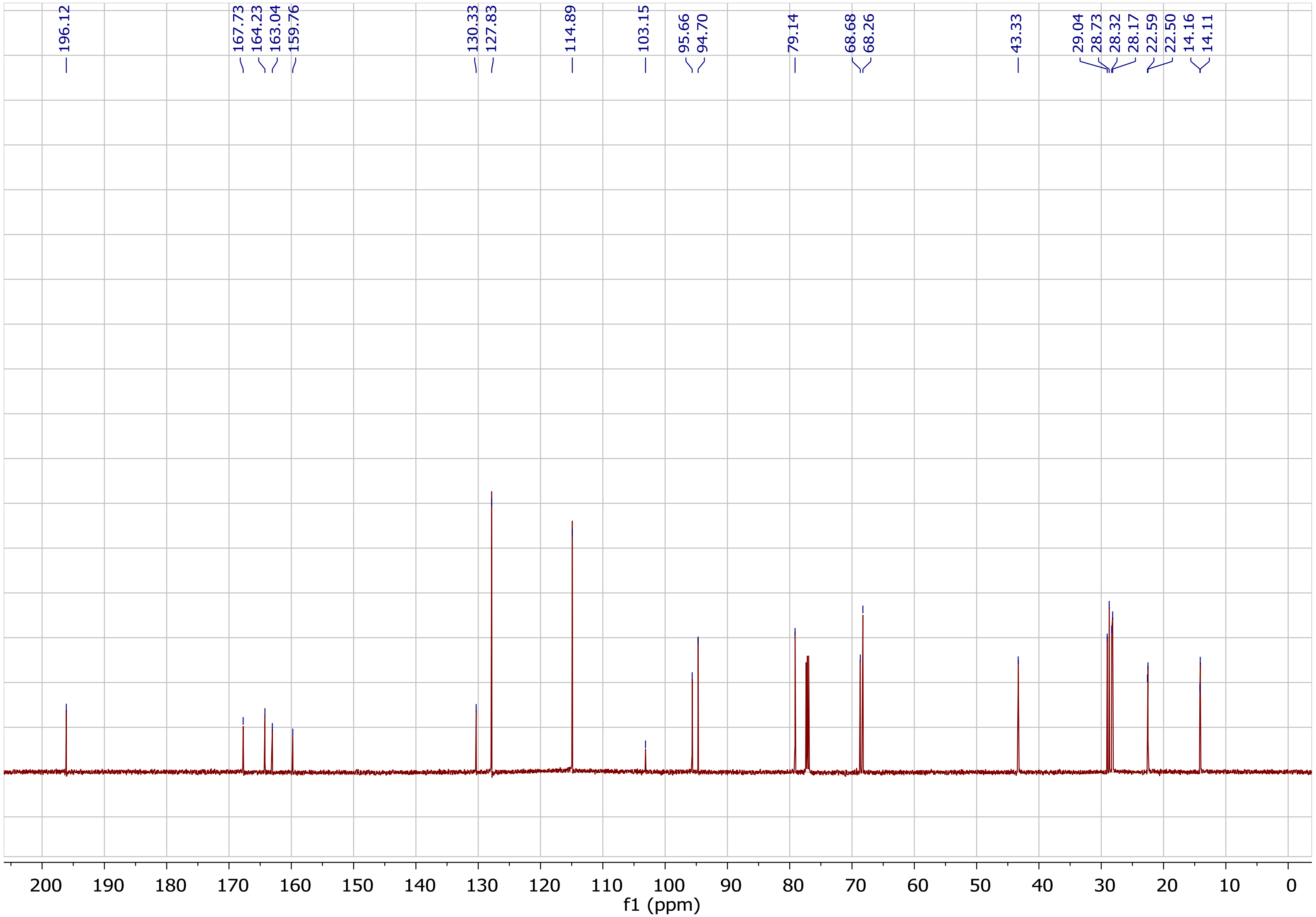

Figure S16. ^{13}C-NMR (150 MHz, $CDCl_3$) spectrum of compound **8a**

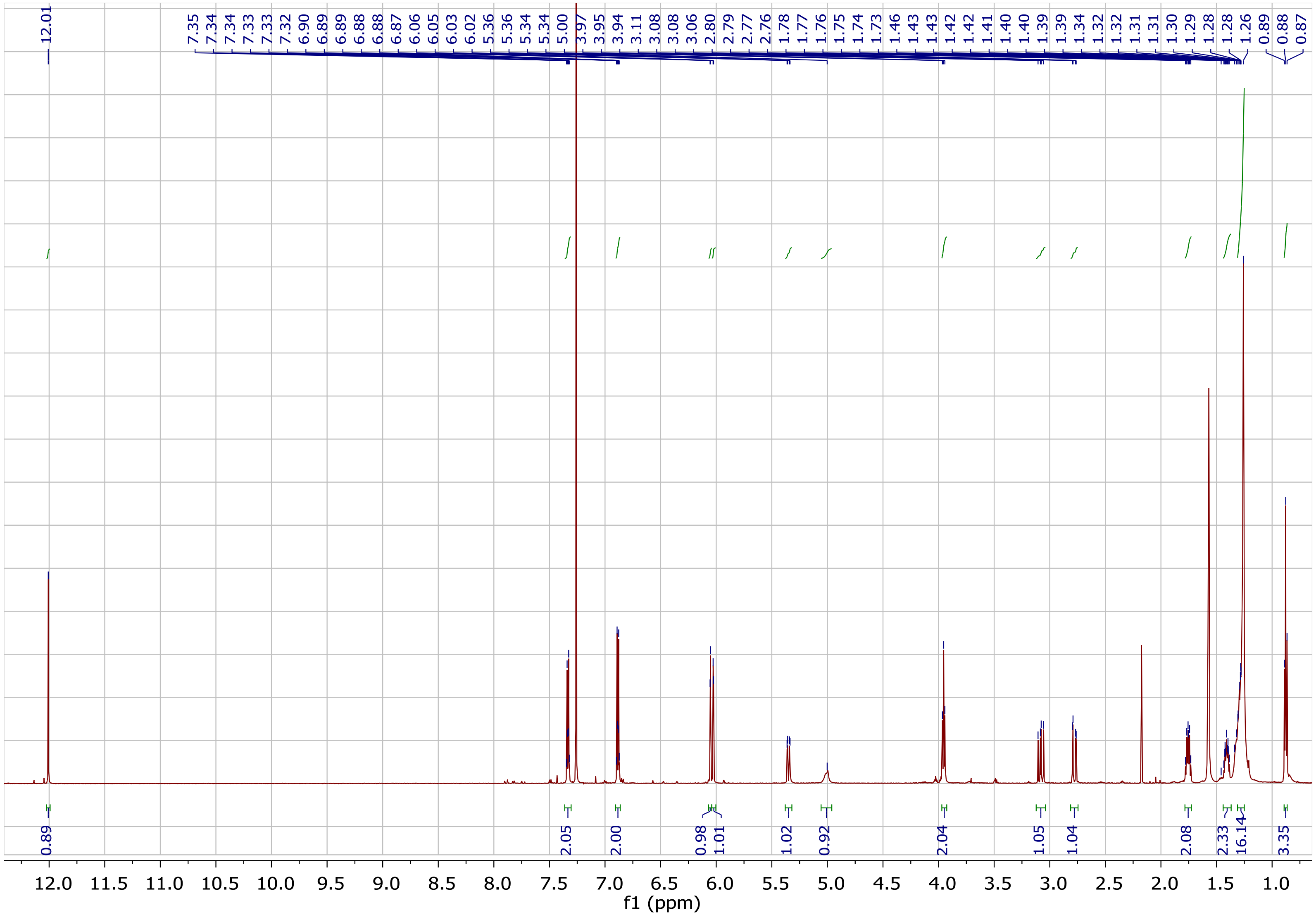

Figure S17. ^{1}H-NMR (600 MHz, $CDCl_3$) spectrum of compound **9a**

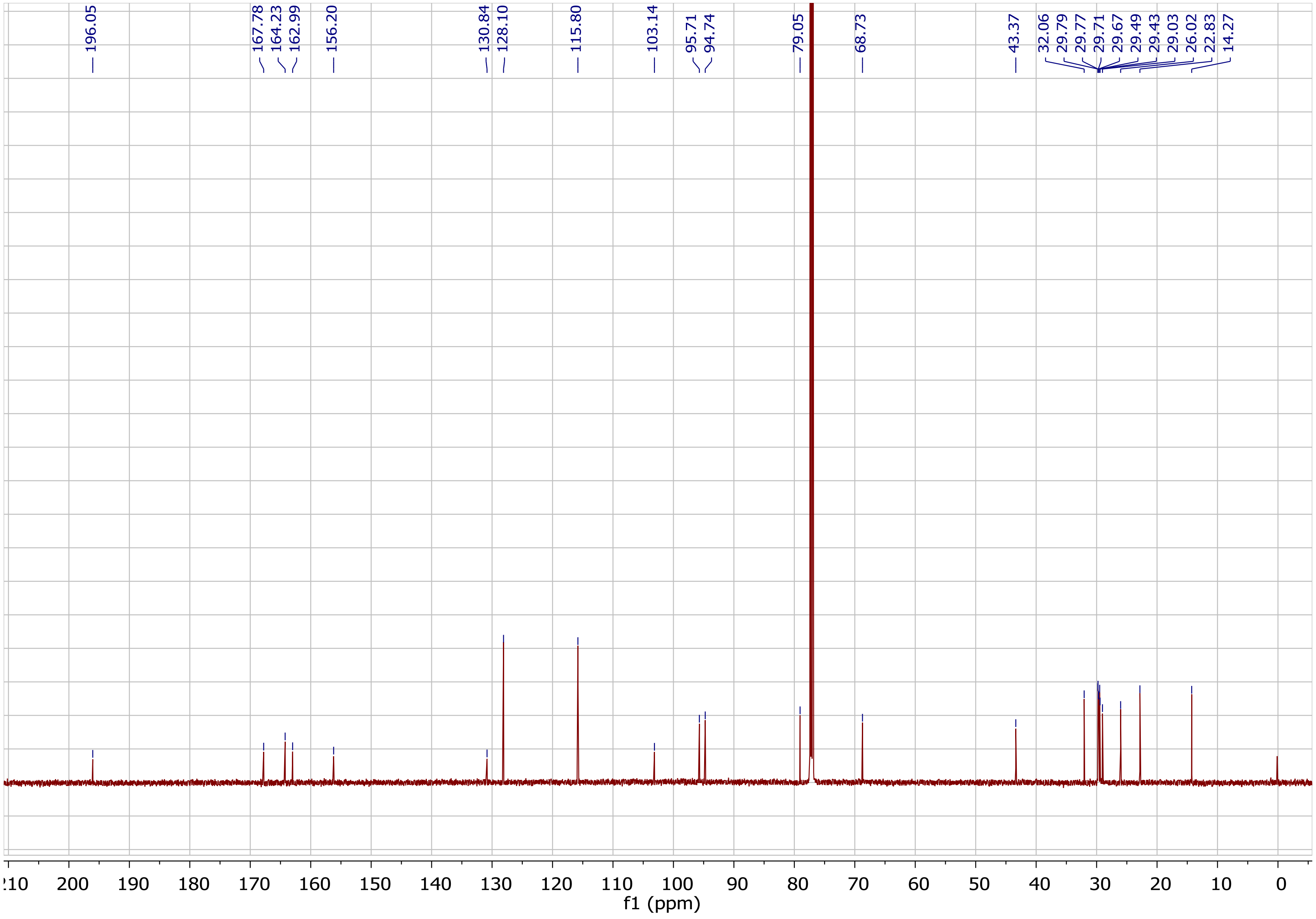

Figure S18. ^{13}C-NMR (150 MHz, $CDCl_3$) spectrum of compound **9a**

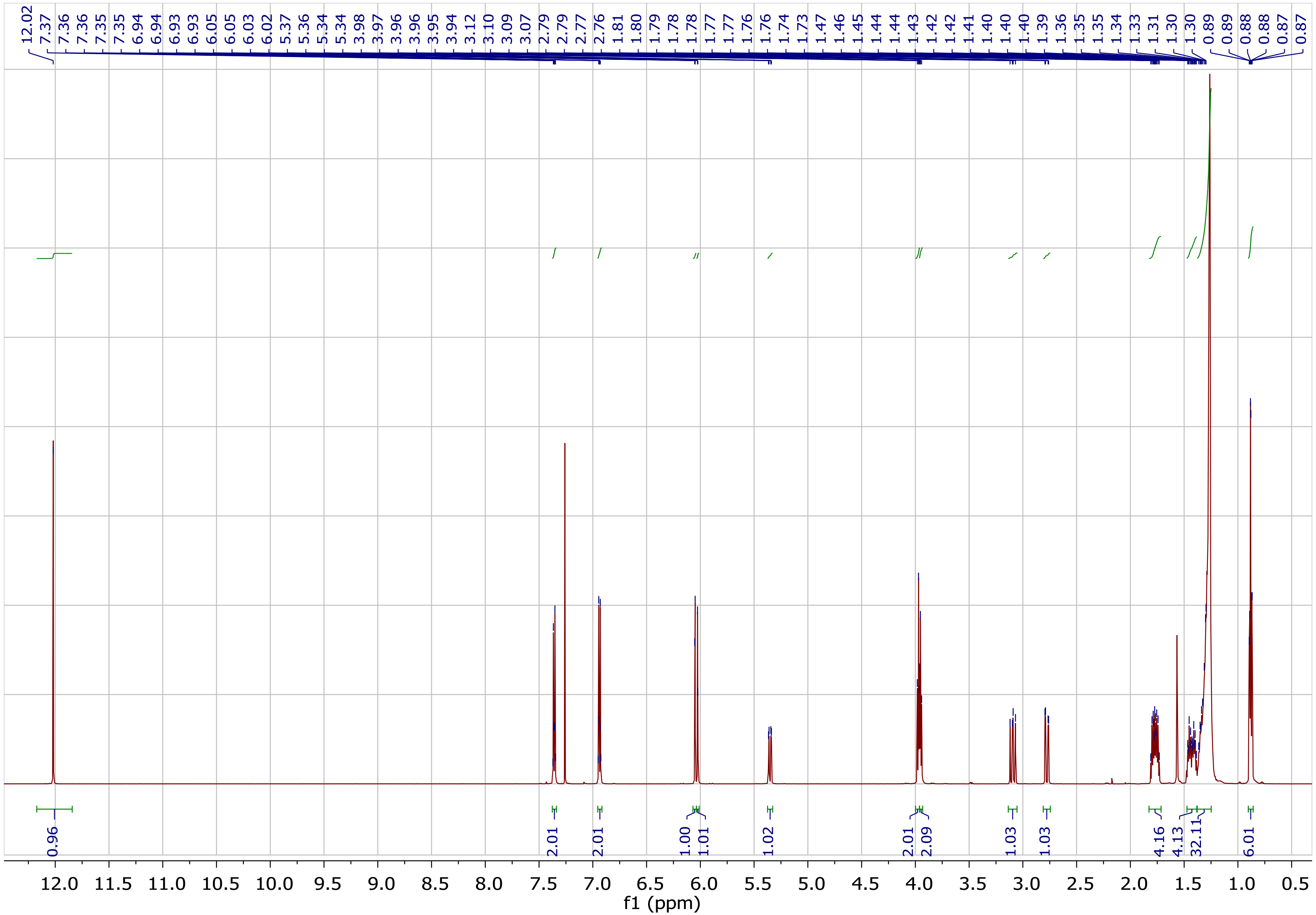

Figure S19. ^{1}H-NMR (600 MHz, $CDCl_3$) spectrum of compound **10a**

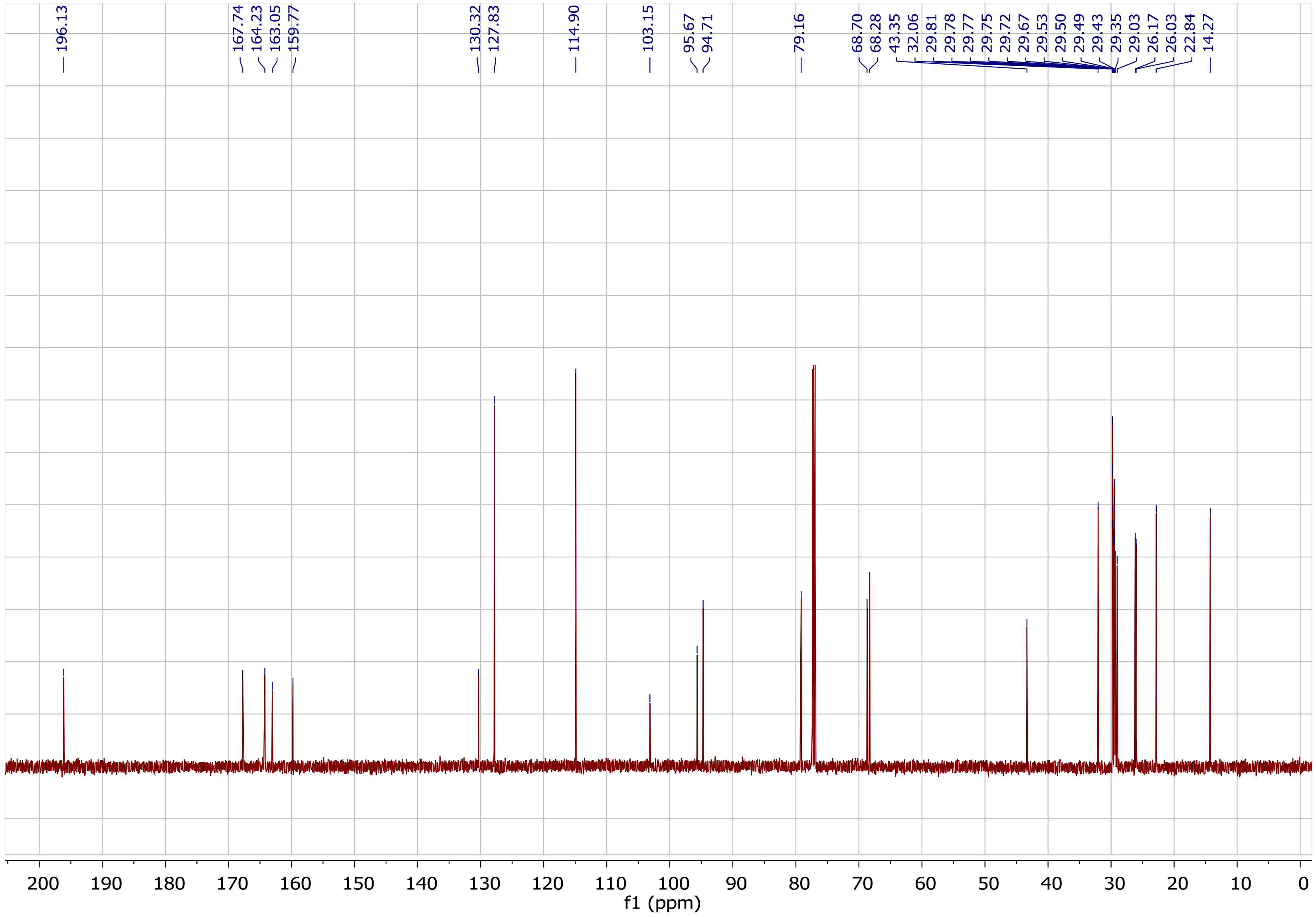

Figure S20. ^{13}C-NMR (150 MHz, $CDCl_3$) spectrum of compound **10a**

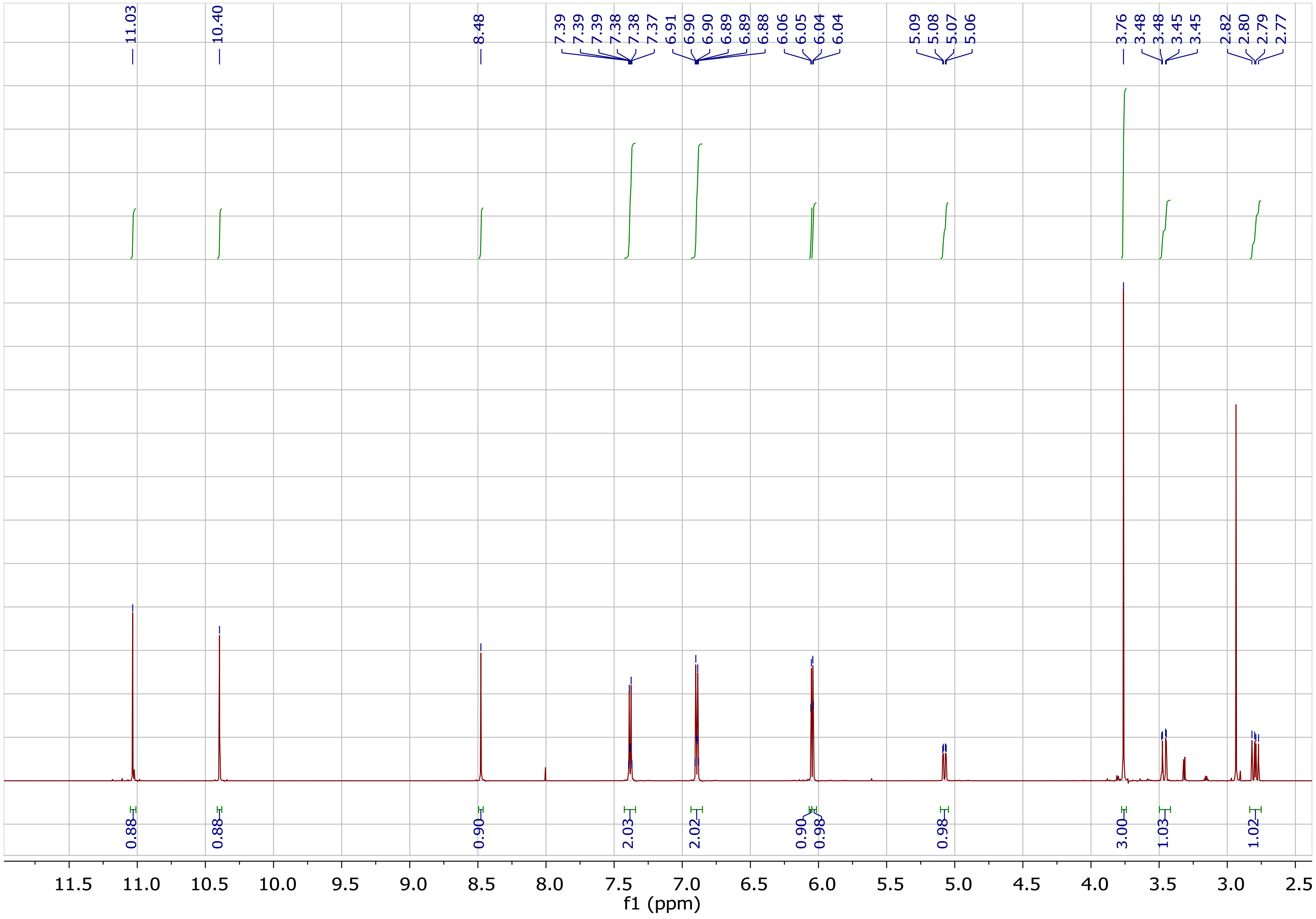

Figure S21. ^{1}H-NMR (600 MHz, Acetone-d6) spectrum of compound **1b**

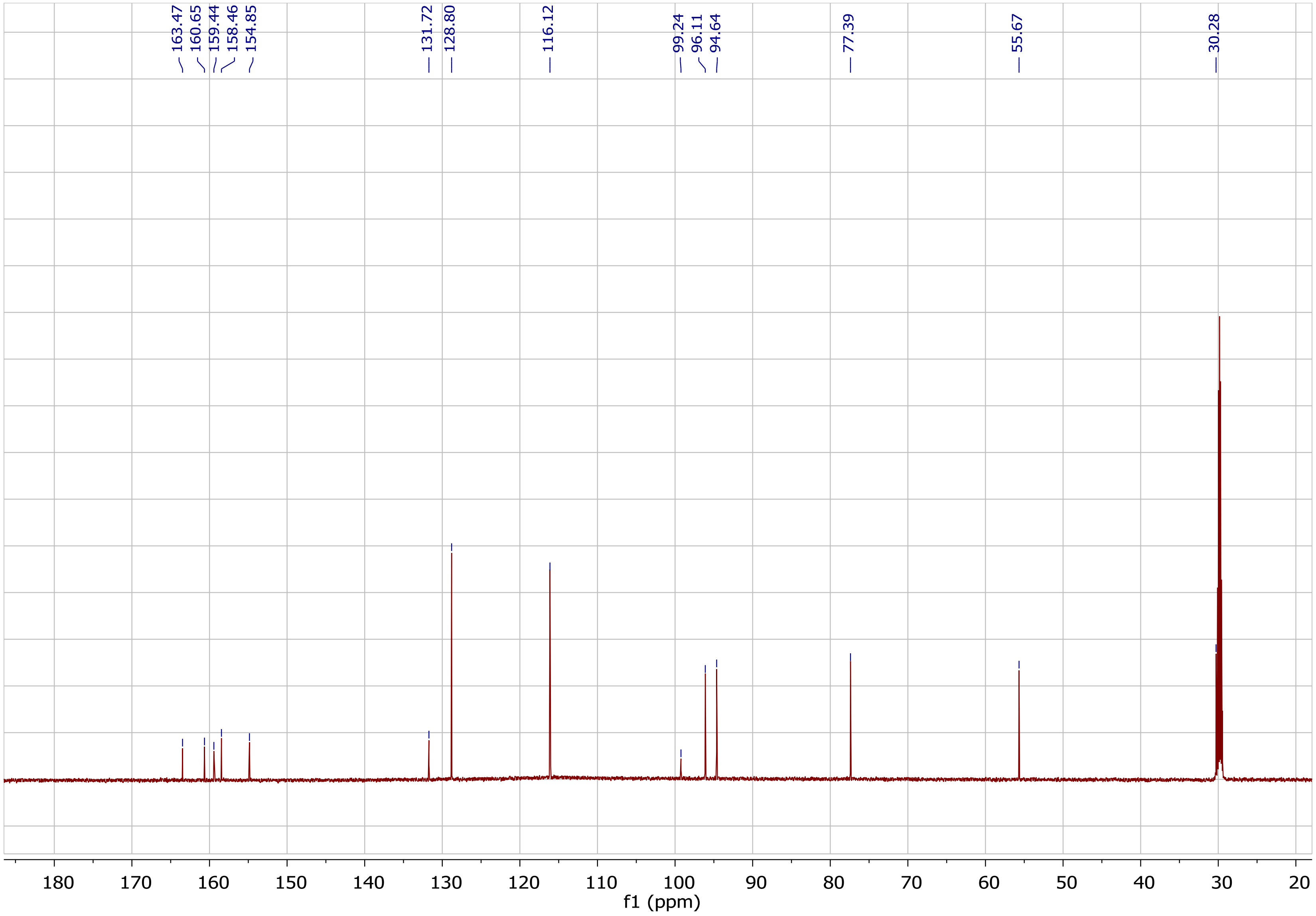

Figure S22. ^{13}C-NMR (150 MHz, Acetone-d6) spectrum of compound **1b**

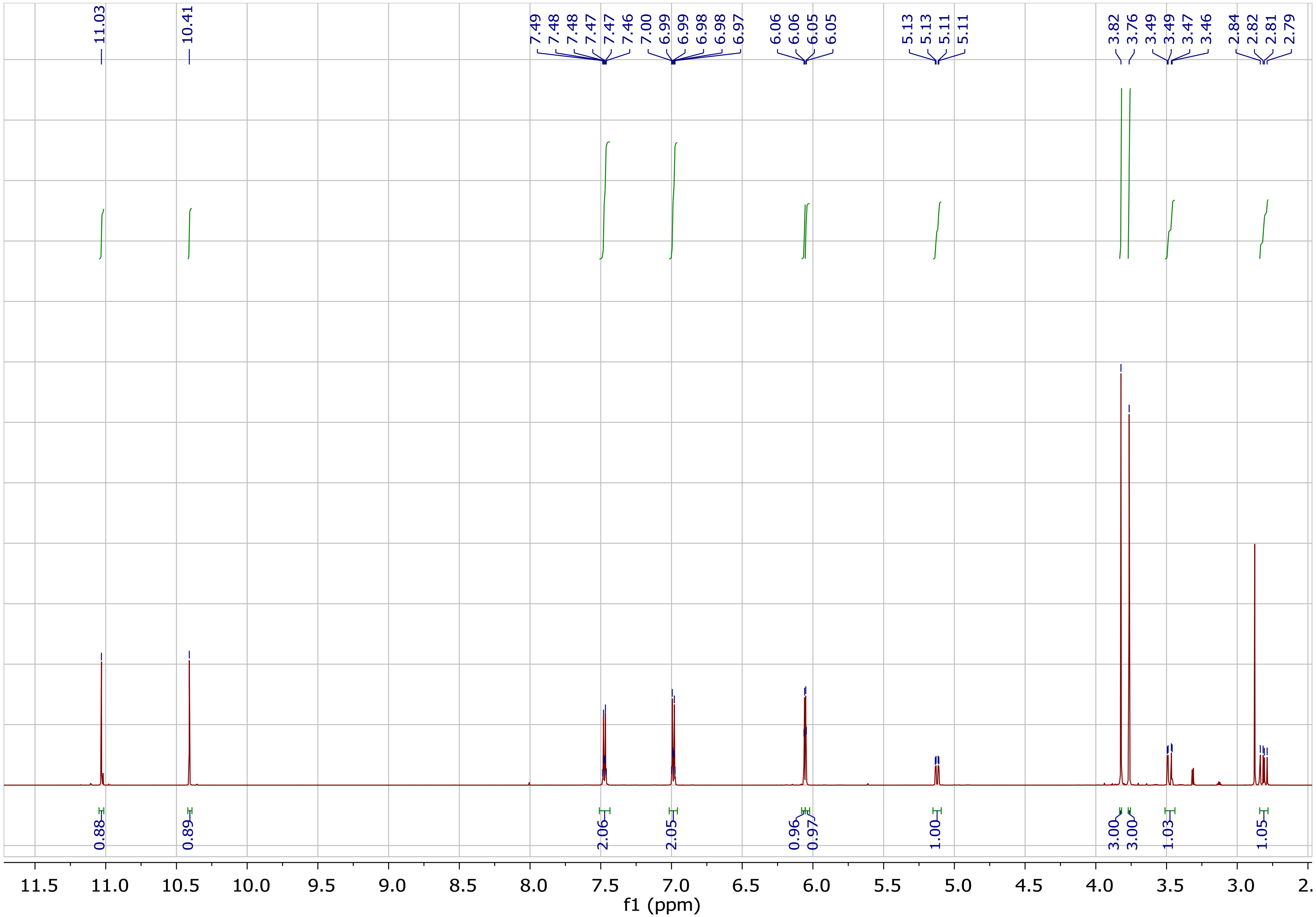

Figure S23. ^{1}H-NMR (600 MHz, Acetone-d6) spectrum of compound **2b**

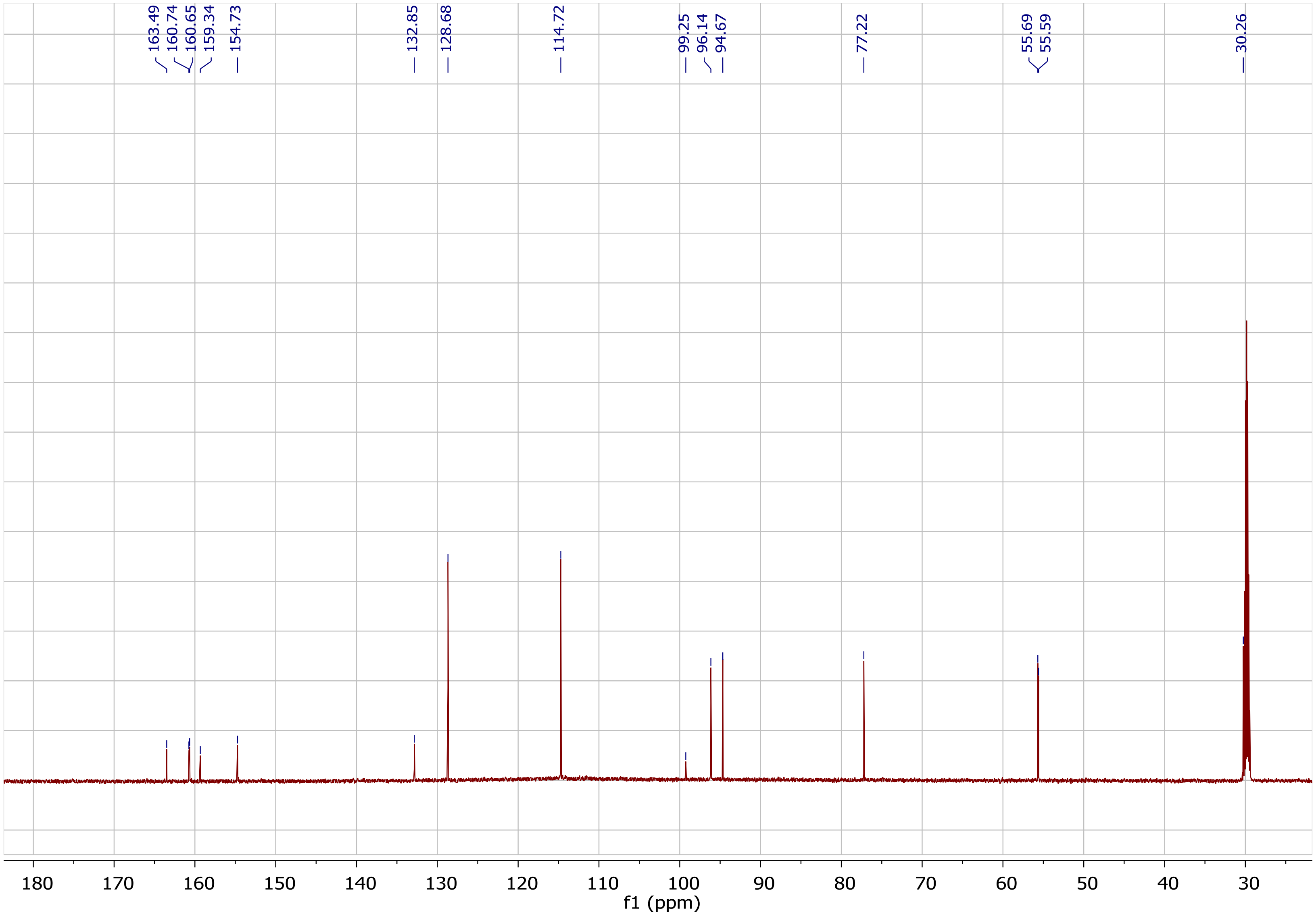

Figure S24. ^{13}C-NMR (150 MHz, Acetone-d6) spectrum of compound **2b**

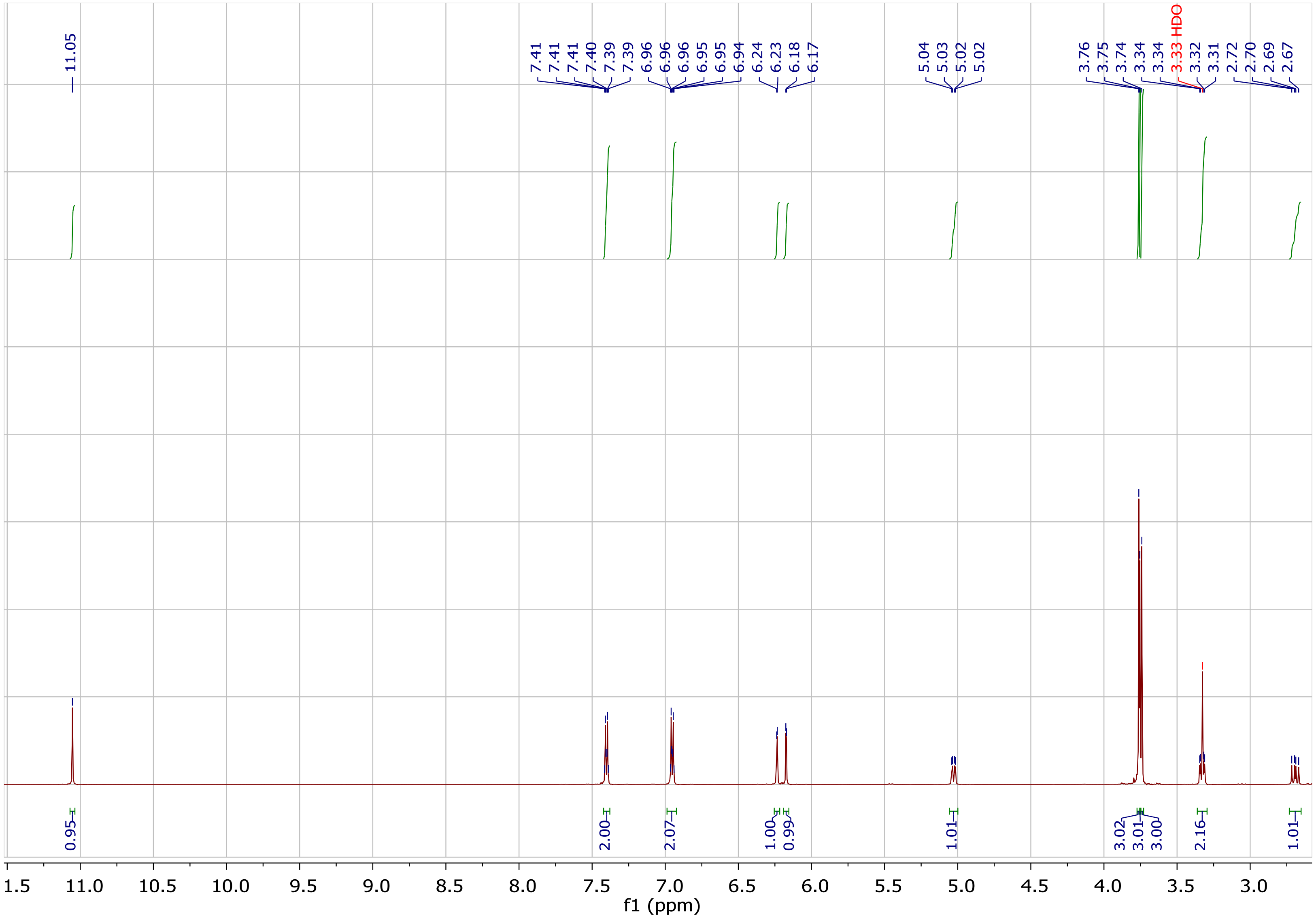

Figure S25. ^{1}H-NMR (600 MHz, DMSO-d6) spectrum of compound **3b**

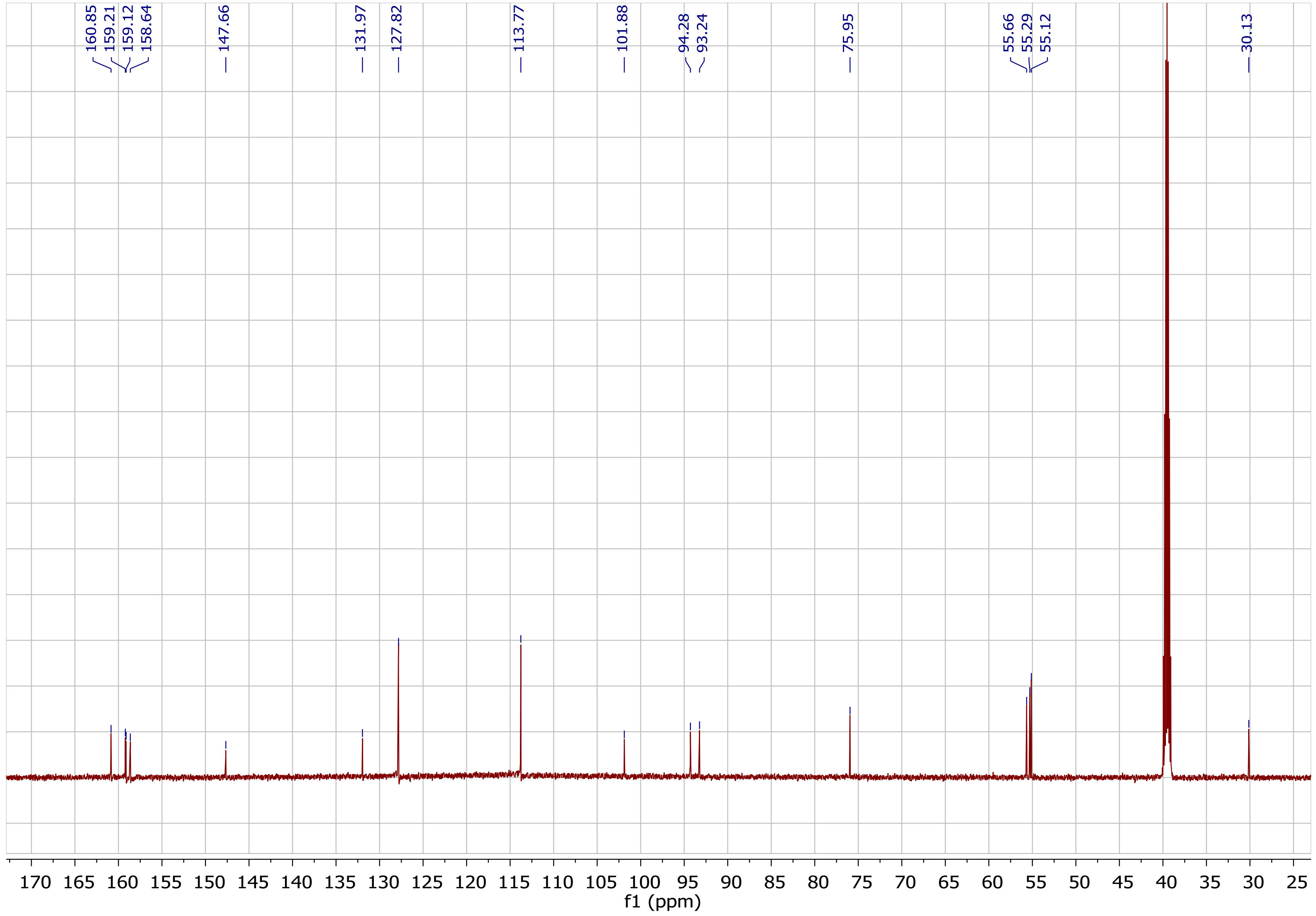

Figure S26. ^{13}C-NMR (150 MHz, DMSO-d6) spectrum of compound **3b**

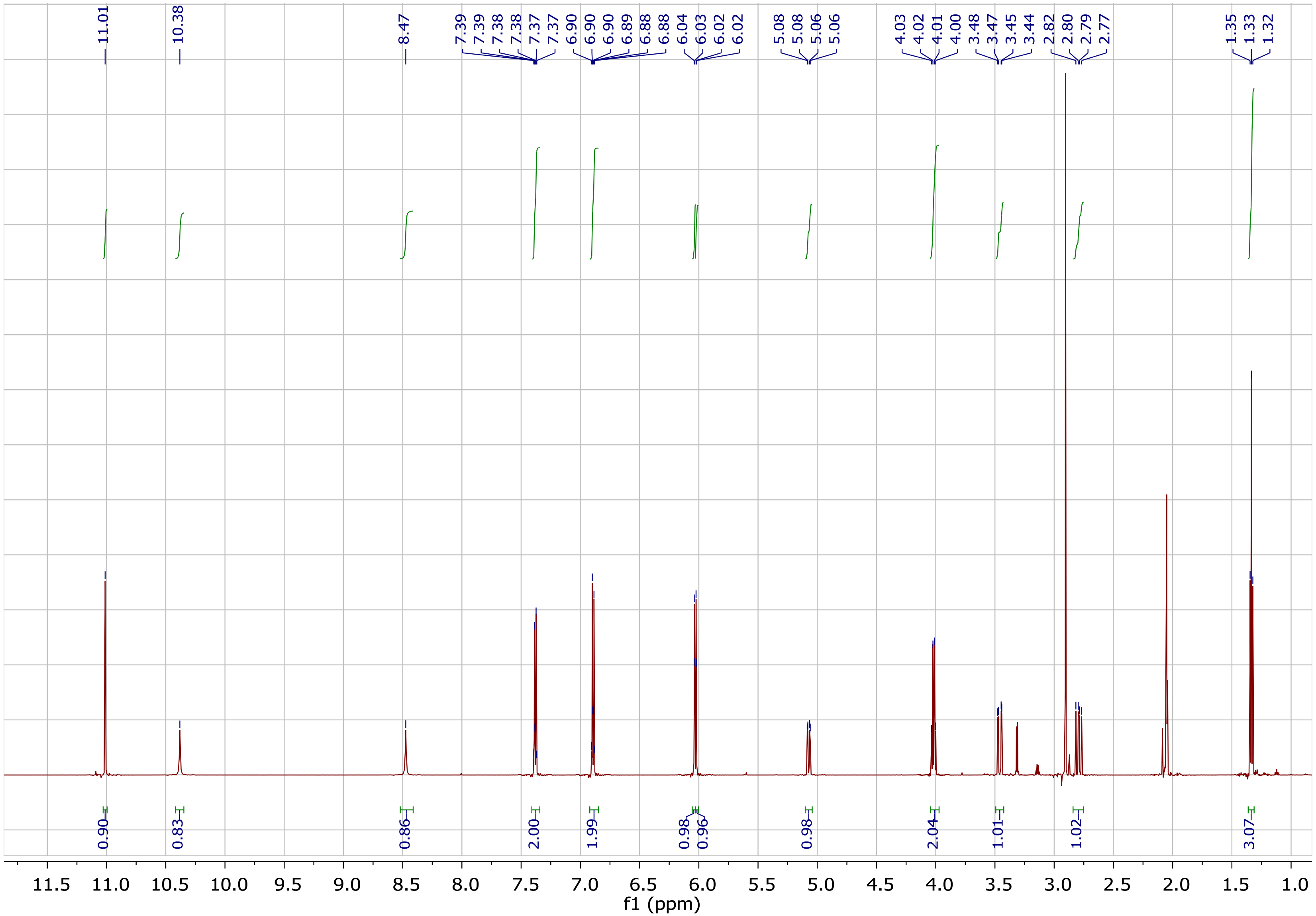

Figure S27. ^{1}H-NMR (600 MHz, Acetone-d6) spectrum of compound **4b**

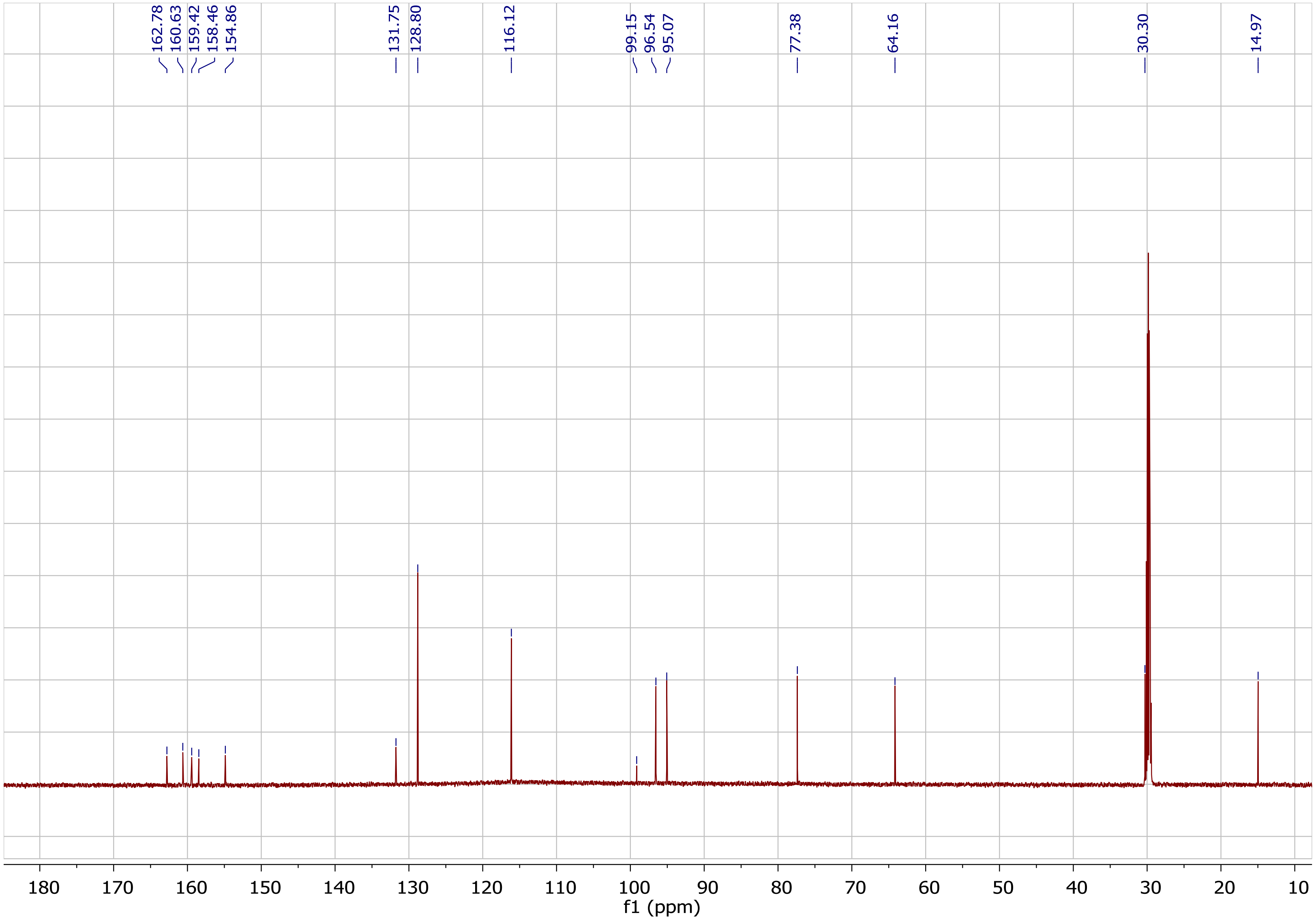

Figure S28. ^{13}C-NMR (150 MHz, Acetone-d6) spectrum of compound **4b**

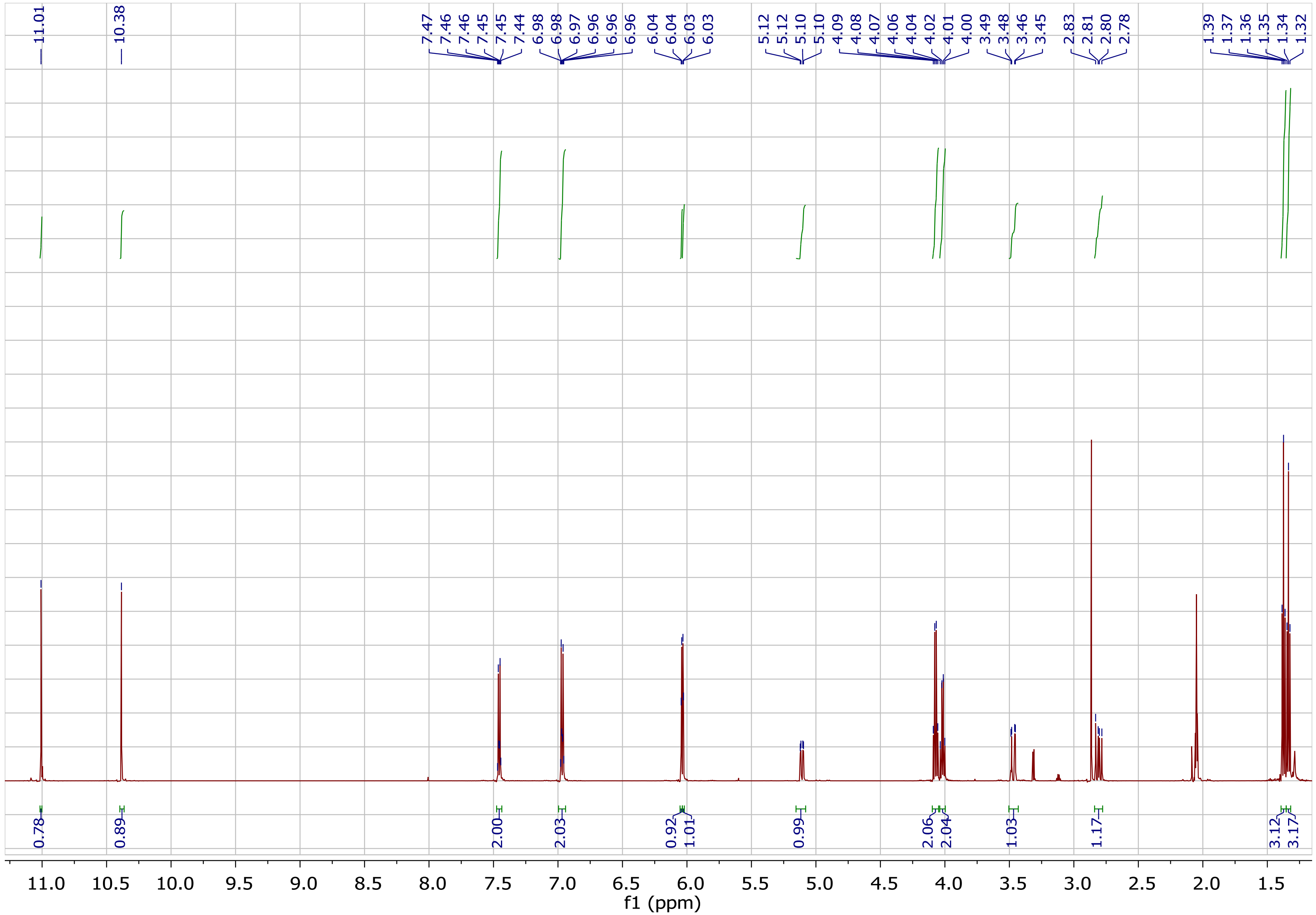

Figure S29. ^{1}H-NMR (600 MHz, Acetone-d6) spectrum of compound **5b**

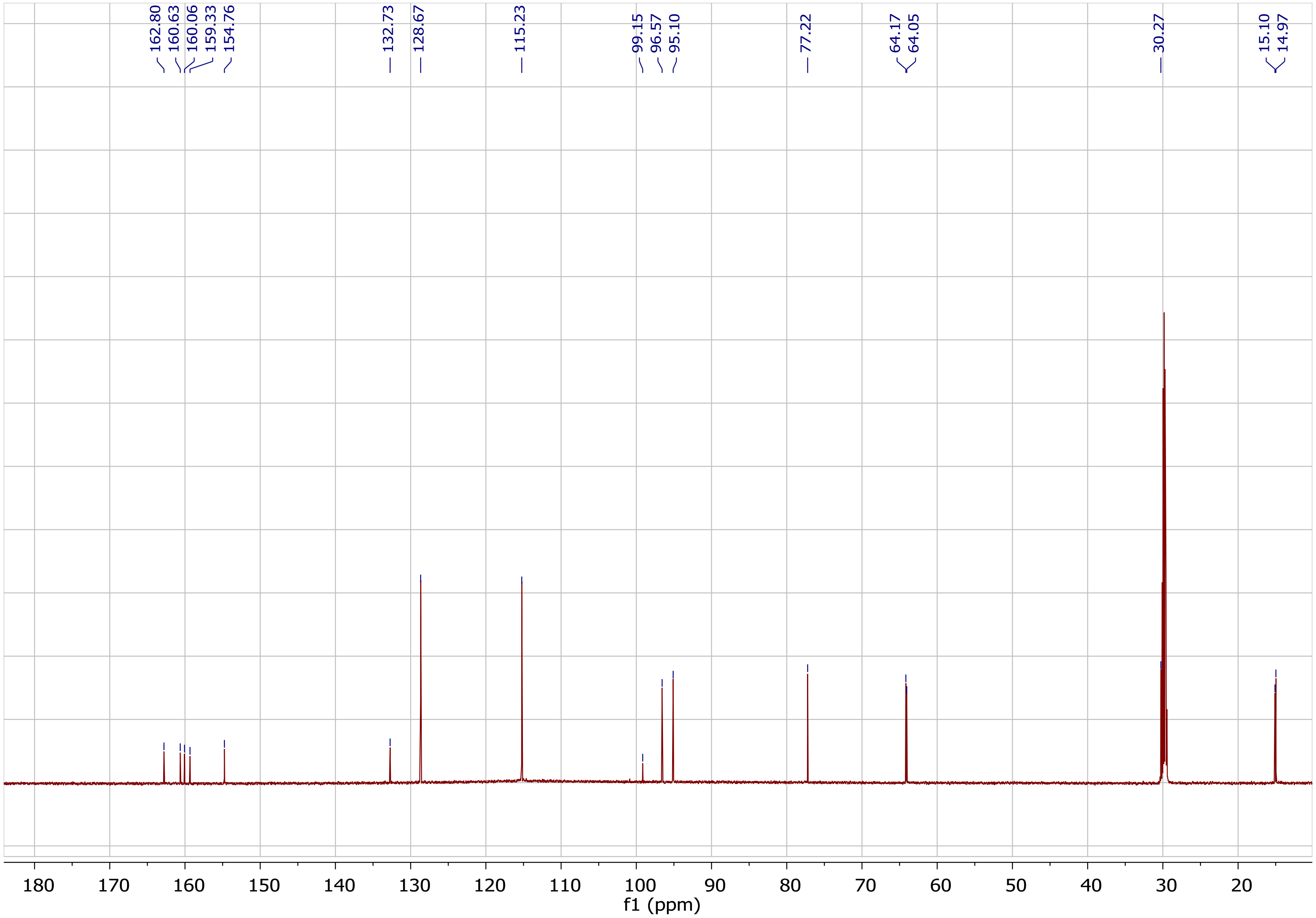

Figure S30. ^{13}C-NMR (150 MHz, Acetone-d6) spectrum of compound **5b**

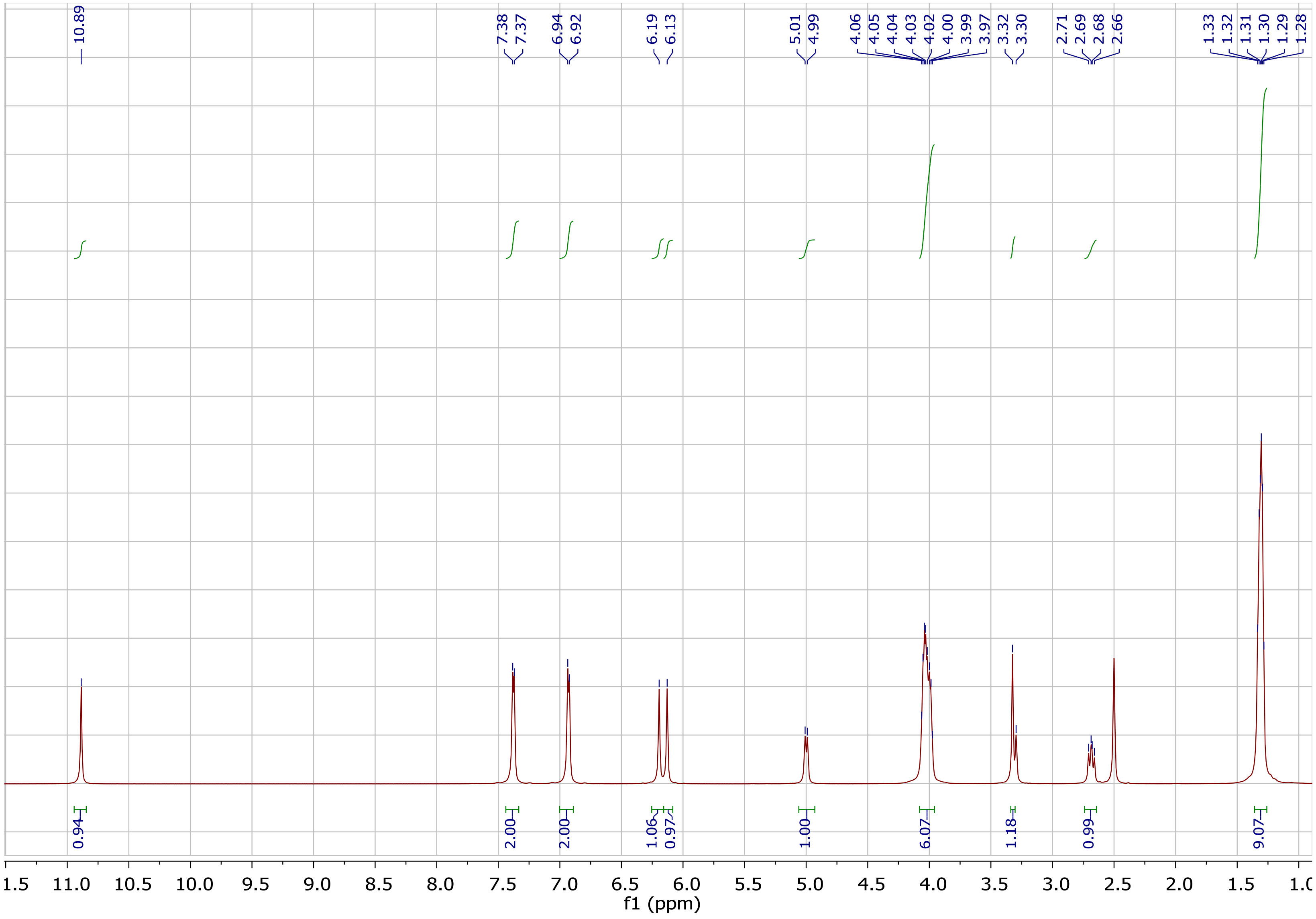

Figure S31. ¹H-NMR (600 MHz, DMSO-d6) spectrum of compound **6b**

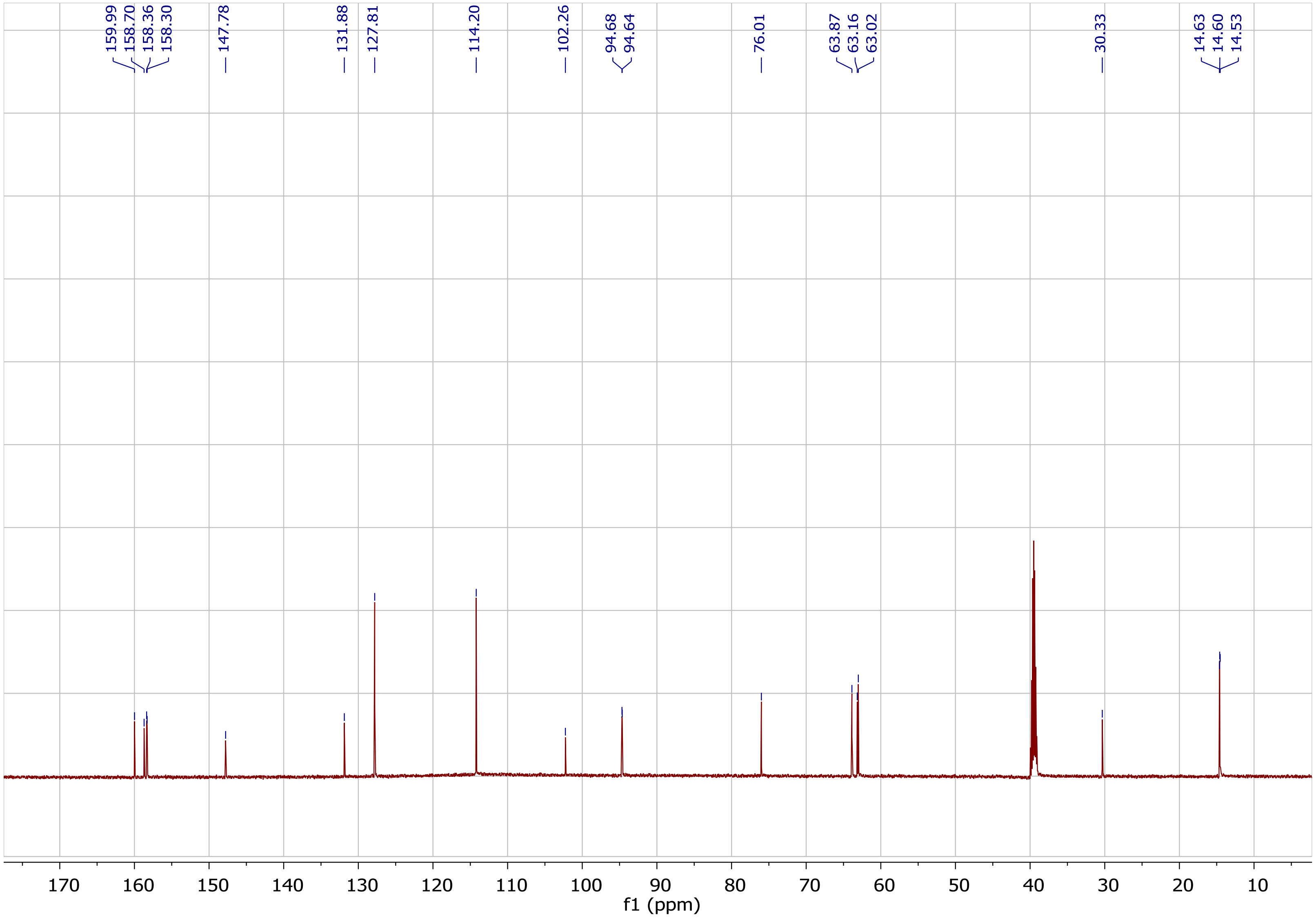

Figure S32. ^{13}C-NMR (150 MHz, DMSO-d6) spectrum of compound **6b**

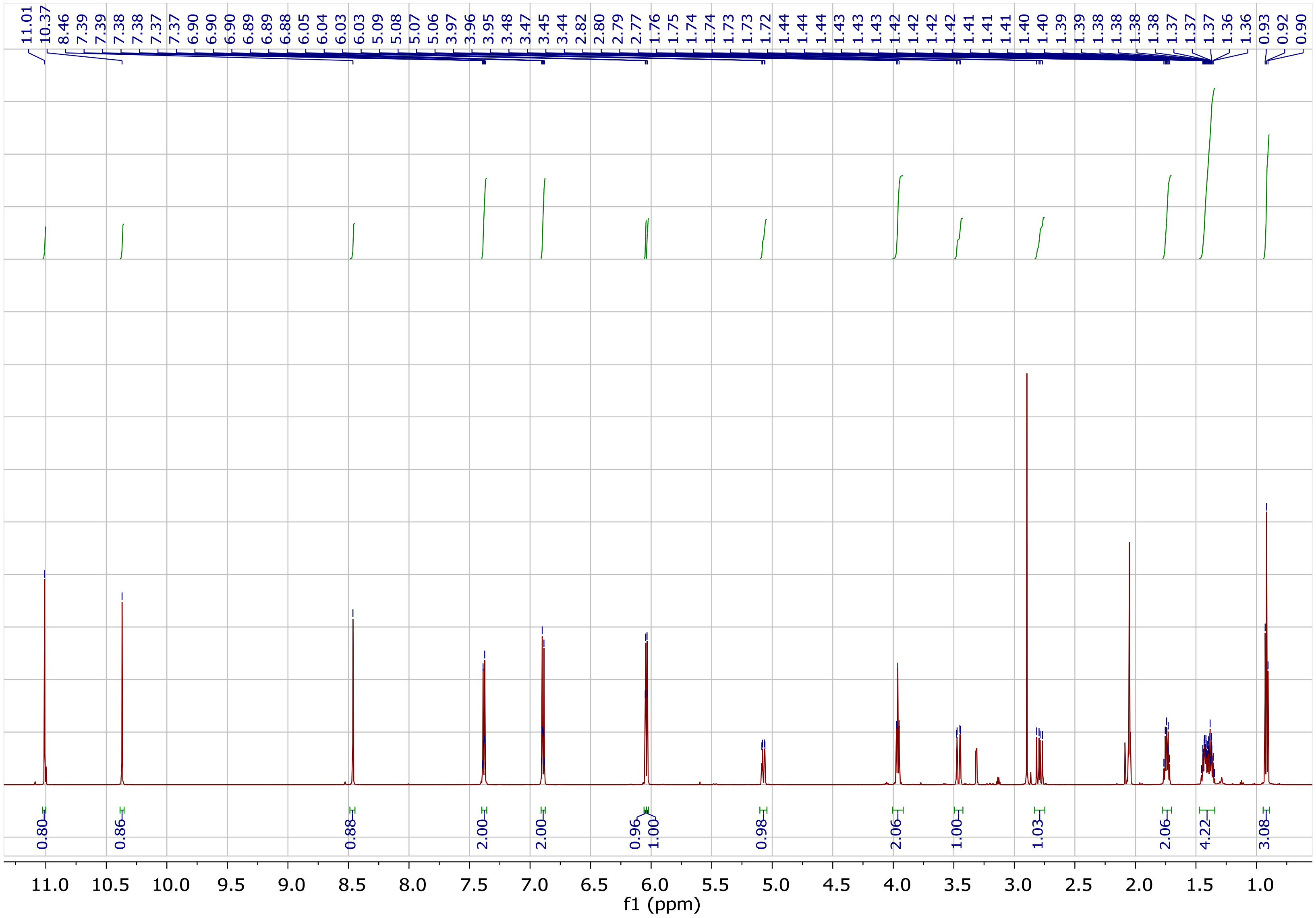

Figure S33. ^{1}H-NMR (600 MHz, Acetone-d6) spectrum of compound **7b**

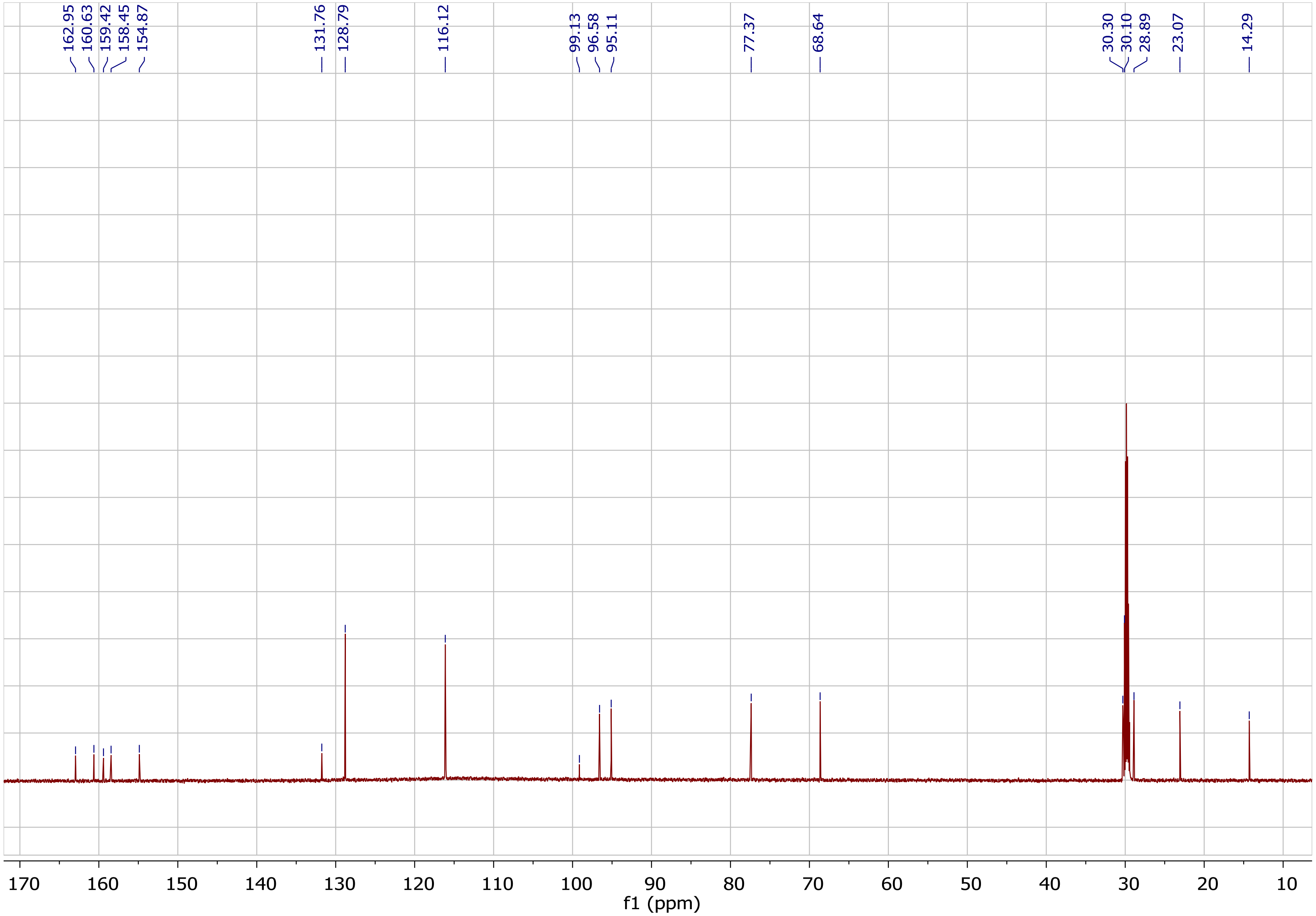

Figure S34. ^{13}C-NMR (150 MHz, Acetone-d6) spectrum of compound **7b**

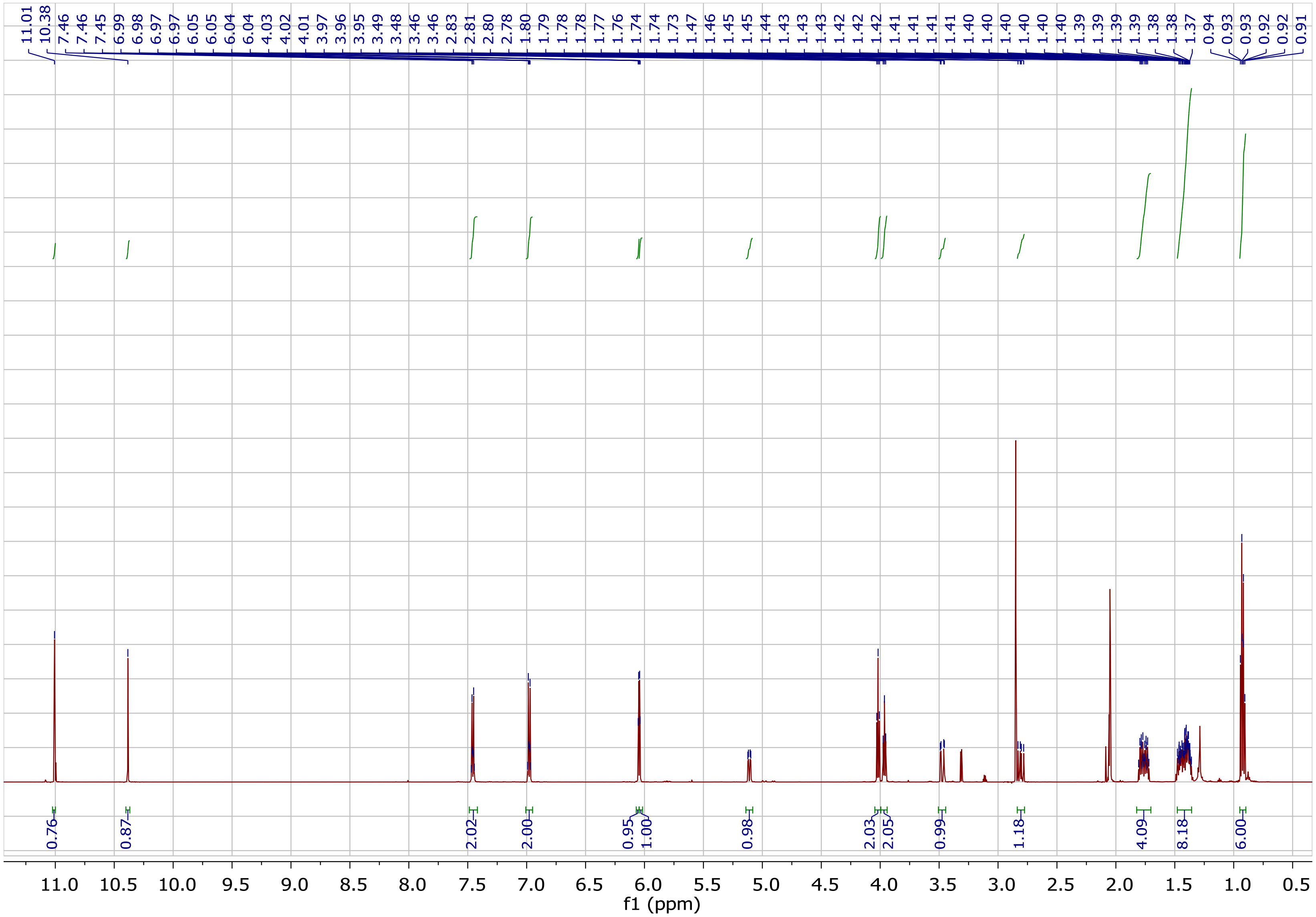

Figure S35. ^{1}H-NMR (600 MHz, Acetone-d6) spectrum of compound **8b**

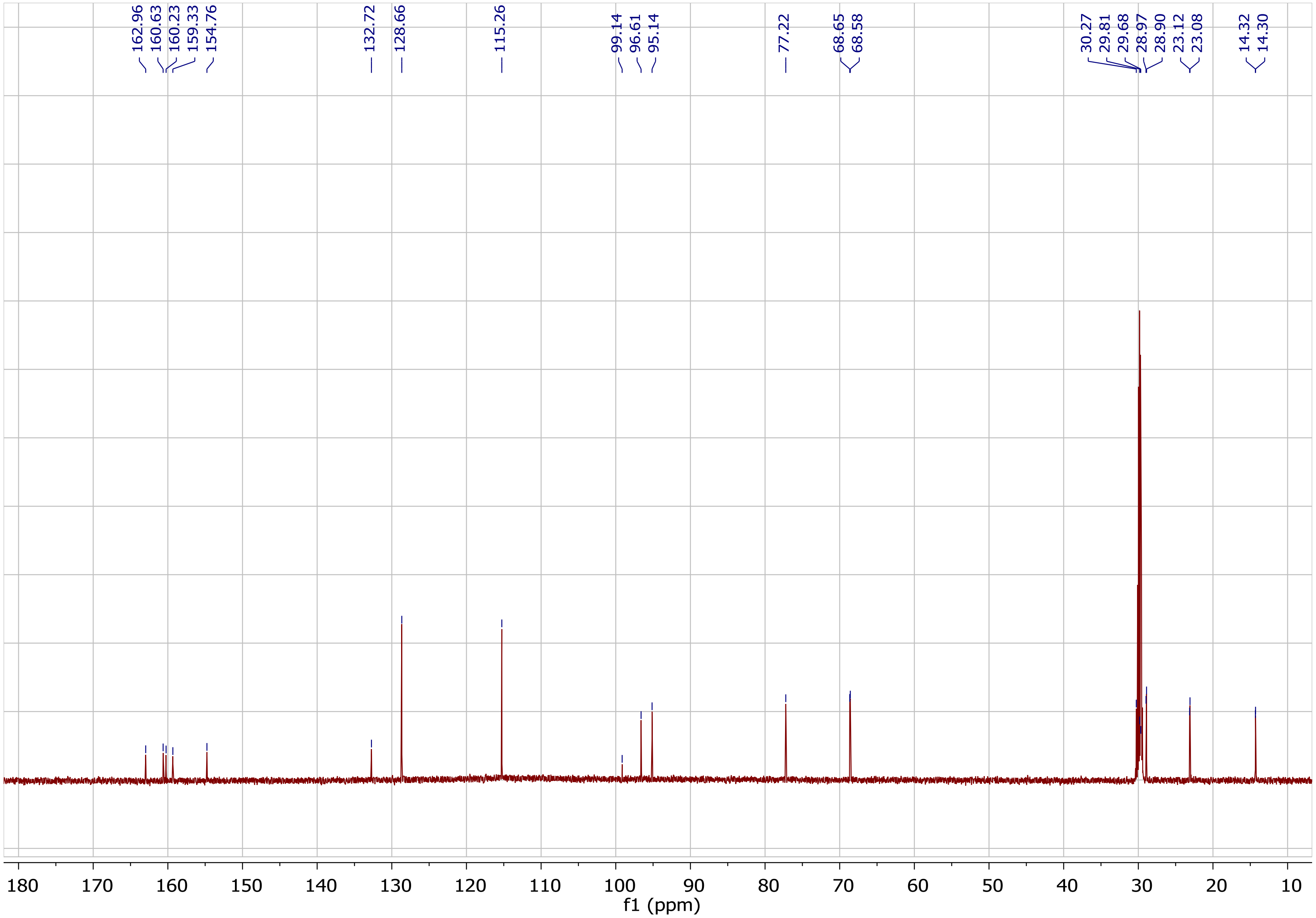

Figure S36. ^{13}C-NMR (150 MHz, Acetone-d6) spectrum of compound **8b**

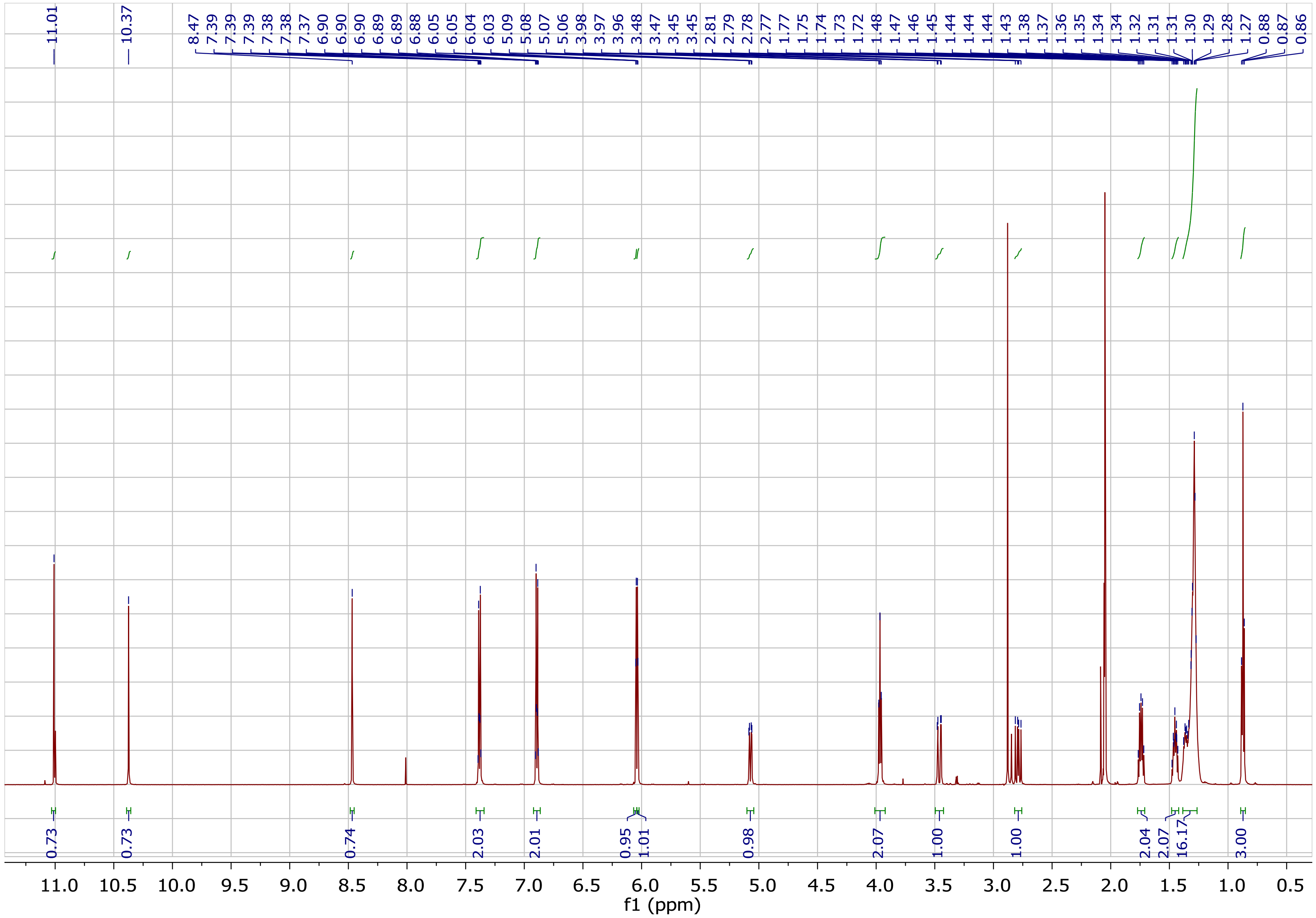

Figure S37. ^{1}H-NMR (600 MHz, Acetone-d6) spectrum of compound **9b**

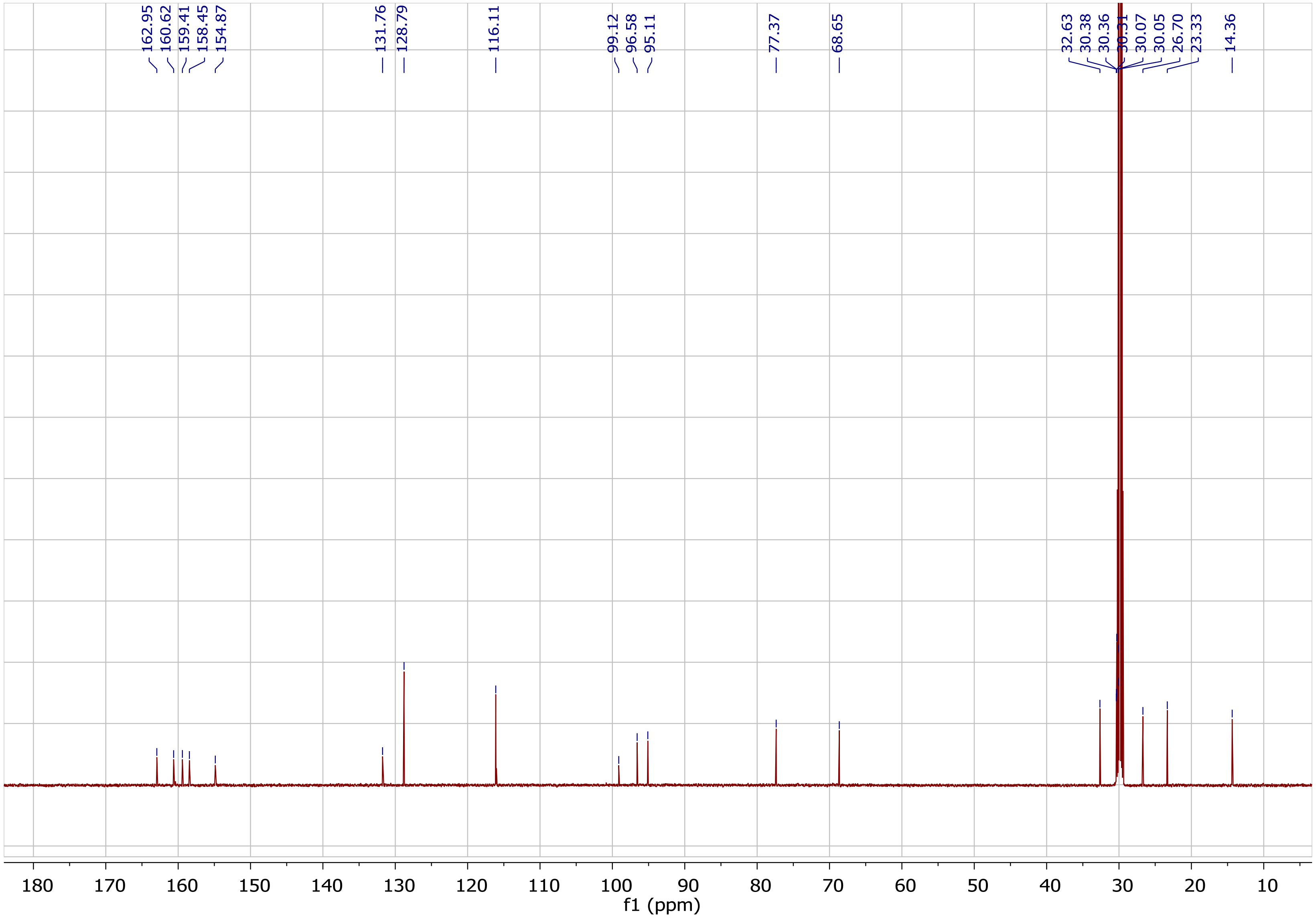

Figure S38. ^{13}C-NMR (150 MHz, Acetone-d6) spectrum of compound **9b**

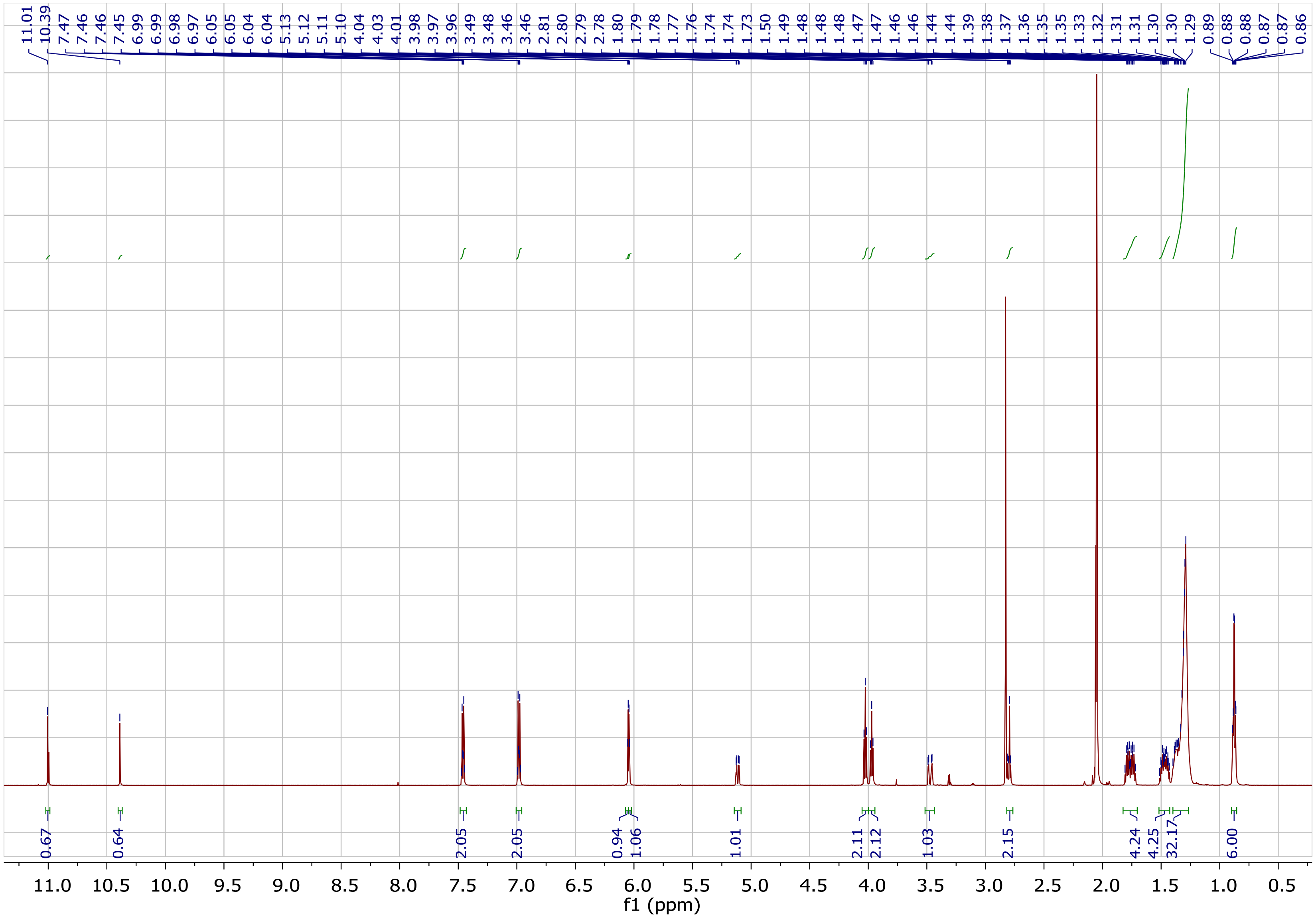

Figure S39. ¹H-NMR (600 MHz, Acetone-d6) spectrum of compound **10b**

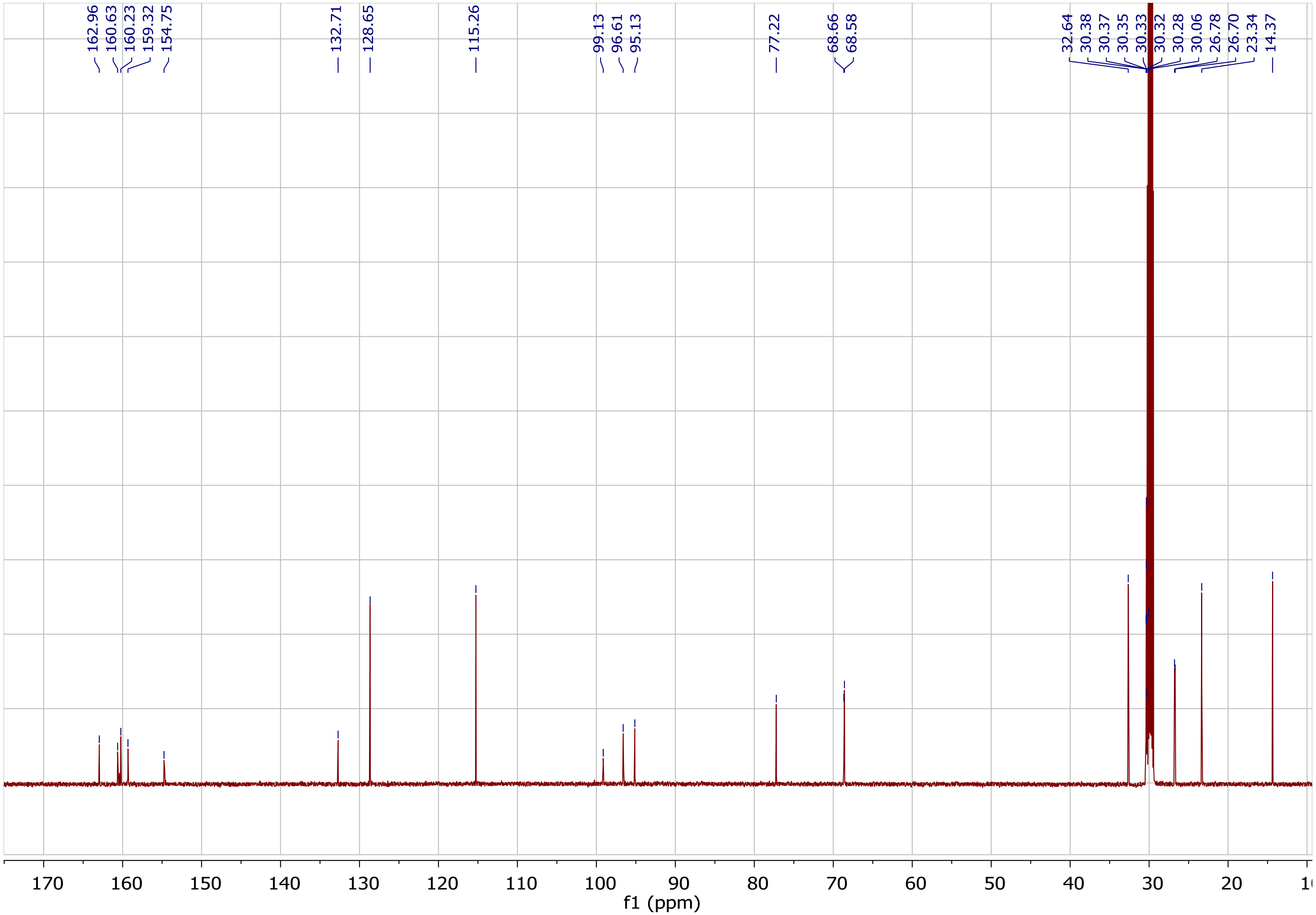

Figure S40. ^{13}C-NMR (150 MHz, Acetone-d6) spectrum of compound **10b**
